# Extending conceptual DFT to include external variables: the influence of magnetic fields†

**DOI:** 10.1039/d1sc07263c

**Published:** 2022-04-04

**Authors:** Robin Francotte, Tom J. P. Irons, Andrew M. Teale, Frank de Proft, Paul Geerlings

**Affiliations:** Research Group of General Chemistry (ALGC), Vrije Universiteit Brussel (VUB) Pleinlaan 2 B-1050 Brussels Belgium; School of Chemistry, University of Nottingham University Park Nottingham NG7 2RD UK Tom.Irons@nottingham.ac.uk andrew.teale@nottingham.ac.uk; Hylleraas Centre for Quantum Molecular Sciences, Department of Chemistry, University of Oslo P.O. Box 1033 Blindern N-0315 Oslo Norway; Research Group of General Chemistry (ALGC), Vrije Universiteit Brussel (VUB) Pleinlaan 2 B-1050 Brussels Belgium fdeprof@vub.be

## Abstract

An extension of conceptual DFT to include the influence of an external magnetic field is proposed in the context of a program set up to cope with the ever increasing variability of reaction conditions and concomitant reactivity. The two simplest global reactivity descriptors, the electronic chemical potential (*μ*) and the hardness (*η*), are considered for the main group atoms H–Kr using current density-functional theory. The magnetic field strength, |**B**|, is varied between 0.0 and 1.0 *B*_0_ = *ħe*^−1^*a*_0_^−2^ ≈ 2.3505 × 10^5^ T, encompassing the Coulomb and intermediate regimes. The carbon atom is studied as an exemplar system to gain insight into the behaviour of the neutral, cationic and anionic species under these conditions. Their electronic configurations change with increasing |**B**|, leading to a piecewise behaviour of the ionization energy (*I*) and electron affinity (*A*) values as a function of |**B**|. This results in complex behaviour of properties such as the electronegativity *χ* = −1/2(*I* + *A*) = −*μ* and hardness *η* = 1/2(*I* − *A*). This raises an interesting question: to what extent are atomic properties periodic in the presence of a magnetic field? In the Coulomb regime, close to |**B**| = 0, we find the familiar periodicity of the atomic properties, and make the connections to response functions central to conceptual DFT. However, as the field increases in the intermediate regime configurational changes of the atomic species lead to discontinuous changes in their properties; fundamentally changing their behaviour, which is illustrated by constructing a periodic table of *χ* and *η* values at |**B**| = 0.5 *B*_0_. These values tend to increase for groups 1–2 and decrease for groups 16–18, leading to a narrower range overall and suggesting substantial changes in the chemistry of the main group elements. Changes within each group are also examined as a function of |**B**|. These are more complex to interpret due to the larger number of configurations accessible to heavier elements at high field. This is illustrated for group 17 where Cl and Br have qualitatively different configurations to their lighter cogener at |**B**| = 0.5 *B*_0_. The insight into periodic trends in strong magnetic fields may provide a crucial starting point for predicting chemical reactivity under these exotic conditions.

## Abstract 2

An extension of conceptual DFT to include the influence of an external magnetic field is proposed in the context of a program set up to cope with the ever increasing variability of reaction conditions and concomitant reactivity.

## Graphical abstract

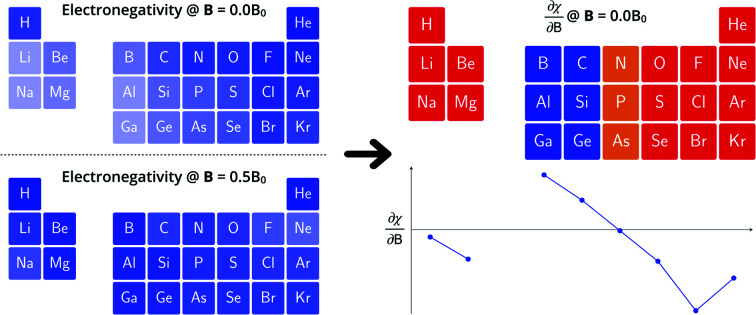

## 1 Introduction

The behavior of atoms and molecules under the influence of external fields has long been of interest to experimental and theoretical physicists and chemists. In particular, the influence of oriented external electric fields on structure and reactivity of molecules was recently examined in detail by both theoretical and experimental chemists.^1,2^ Evidence was presented, in the pioneering work by Shaik and coworkers,^2^ that oriented external electric fields could potentially be used to exert unprecedented control over chemical reactivity, offering a plethora of new synthetic tools for organic, metallo-organic and bioorganic chemists to explore chemical space.^3^ In short, “they are expected to be novel effectors of chemical change”.^2^

The influence of (strong) magnetic fields on the other hand has received less attention. Theoretical studies on atomic systems have been motivated by the astrophysical discovery of strong magnetic fields on white dwarf and neutron stars, with fields of the order of 10^2^–10^5^ T and 10^7^–10^9^ T, respectively.^4^ The energies of the most important low-lying states of a number of light atoms have been studied as a function of magnetic field strength.^5–13^ These studies have focused on determining how the electronic configuration of the ground state changes as the magnetic field increases, and how the field distorts the electron density. Calculations on molecular systems are technically more challenging since lower symmetries make it more difficult to apply accurate mesh-based approaches and finite basis set techniques must be adapted to allow for complex orbitals, whilst ensuring that the calculation of energies and physical observables remain independent of the gauge-origin associated with the vector potential describing the magnetic field. This challenge was addressed in 2008 by Tellgren *et al.*^14^ with the development of non-perturbative calculations using London atomic orbitals^15^ for general molecular systems. In 2012, Lange *et al.* extended this approach to full-configuration interaction theory,^16^ revealing a new bonding mechanism (“perpendicular paramagnetic bonding”) occurring in magnetic fields of the order of 10^5^ T. This leads to exotic new chemistry, for example the ββ-component of the triplet state of the hydrogen molecule, unstable to dissociation under normal conditions, not only becomes bound but also becomes the ground state at high field strengths.^16^

Recently, the influence of an external mechanical force^17,18^ has also been considered. The field of mechanochemistry refers to unusual chemical reactions induced by mechanical energy. It is the molecular analogue of grinding on the macroscopic scale. A prominent example of this type of reaction is the circumvention of the Woodward–Hoffmann rules for the electrocyclic ring opening of cyclobutene.^19^

In the context of this ever increasing variability of reaction conditions and concomitant reactivity, theories that aim to provide qualitative or quantitative insight into aspects of reactivity should be broadened to account for the effect of reaction conditions. This inspired some of the present authors to embark on a program to extend conceptual density functional theory (DFT).^20–23^ Central to the original conceptual DFT approach,^20,21,24^ developed originally in the 1980s by Parr and coworkers, is the functional *E* = *E*(*N*, *v*) for a given atom, molecule or solid state system, and its variation under perturbations of the system with respect to its number of electrons *N* and/or external potential *v* (*i.e.* the potential felt by the electrons due to the nuclei). These are precisely the perturbations experienced by a given atom or molecule at the onset of a chemical reaction. The various derivatives of the energy *E* with respect to *N* and/or *v* can be readily identified as response functions, quantifying the response of a system to the respective perturbation at the onset of a chemical reaction, hence their collective name of “reactivity descriptors”.

Examples such as the electronic chemical potential 
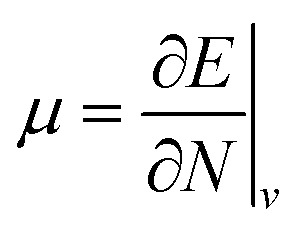
 (ref. 24) (identified as the negative of Mulliken's electronegativity, *χ*),^25^ the chemical hardness 
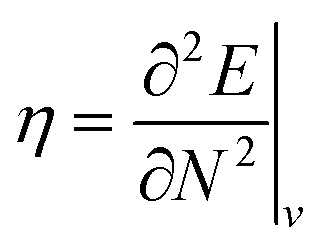
,^26^ the density 
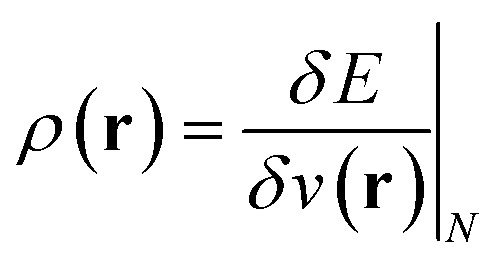
,^20,22^ the Fukui function 
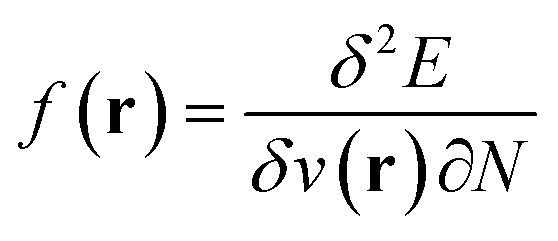
 (ref. 27) and the linear response function 
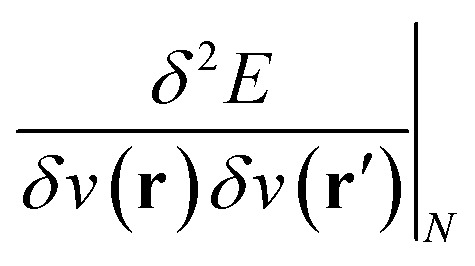
 (ref. 20 and 28) are well documented and their significance has been highlighted extensively in the literature on fundamental and applied aspects of conceptual DFT. Higher order derivatives have also been addressed.^29–31^ The number of variables considered in the energy functional has been extended to include, for example, spin^32,33^ and temperature.^34,35^ External mechanical forces were also introduced recently by some of the present authors,^36–38^ followed by the inclusion of external electric fields^39^ after pioneering work by Chattaraj and Pal.^40–42^

These additional variables not only increase the scope of reaction conditions that conceptual DFT can be applied to, but also increase the number of relevant response functions that can be calculated. This significantly extends the “response function tree” (see for example ref. 22), since the new descriptors are intertwined with the more conventional electric field related atomic and molecular response functions, such as the (permanent) dipole moment, the polarizability and the first hyperpolarizability. Variations of the electronic chemical potential, hardness, electron density and Fukui function with external fields in different orientations were recently calculated and analyzed for simple diatomic molecules (the dihalogens F_2_, Cl_2_, Br_2_, I_2_) and H_2_CO in ref. 39.

A natural extension of this work on electric fields is the inclusion of a magnetic field and the reactivity descriptors that arise. Some early work in this direction has been done by Chattaraj and coworkers. They concentrated on the second-order derivative of *E* with respect to **B**, the magnetizability, and identified the magnetizability as a measure of softness (the inverse of the hardness),^43^ proposing a minimum magnetizability principle^44^ in analogy to the well-known maximum hardness^45^ and the related minimum polarizability^46^ principles.

In this work we will provide a systematic study of the two simplest global (*i.e.***r**-independent) response functions, the electronic chemical potential or (minus) the electronegativity and the hardness, in the presence of an external magnetic field, for atoms belonging to the main group elements of the first four rows of the periodic table (H–Kr). In view of the basic formulas discussed in Section 2, when studying the field dependence of electronegativity and hardness, the computation of atomic ionization energies and electron affinities as a function of the magnetic field strength is a central requirement. A systematic study of these quantities for different field strengths over the periodic table has, to the best of our knowledge, never been presented in the literature. Early work on the calculation of atomic energies in the presence of magnetic fields was focused on hydrogen and helium (for reviews, see ref. 5 and 11). Much less work has been undertaken to study atoms with more than two electrons in magnetic fields. An important early contribution to this field was made in 1996 by Jones, Ortiz and Ceperley,^6^ who published a study at the Hartree–Fock level for a series of light atoms/ions encompassing H, H^−^, He, Li and C, pinpointing the dependence of the ground state configuration on the field strength. Similar studies, with increasing levels of theory were published in later years for Li, Be, and B by Ivanov, Schmelcher and coworkers.^7–10,12,13^ In some of these studies, both the neutral atom and its cation (with possibly different evolution of their electronic configurations with field strength) were examined, allowing the study of the ionization energy as a function of magnetic field strength for Li,^8,12^ Be,^10,13^ and B.^9^ A particularly interesting study in this series from a chemist's point-of-view is that by Ivanov and Schmelcher,^47^ who published a very detailed Hartree–Fock level investigation of the carbon atom, in magnetic fields ranging from 0 to 2.23 × 10^9^ T, clearly revealing the appearance of chemically counter-intuitive ground state configurations that gradually maximise the number of β electrons and the total angular momentum, which becomes increasingly favourable with increasing magnetic field strength due to the spin Zeeman interaction, up to fully spin-polarized configurations in the very high field regime. These studies highlight how the ground state electronic configuration is very sensitive to the field strength. As a result, different chemical behavior would be expected in various field strength domains driven by these configuration changes.

In this work, we investigate the extent to which periodicity of chemical properties is preserved in the presence of strong magnetic fields. First, we evaluate *χ* and *η* over a wide range of field strengths varying from 0 to 1 *B*_0_ (*B*_0_ = *ħe*^−1^*a*_0_^−2^ ≈ 2.3505 × 10^5^ T), which encompasses the Coulomb and intermediate regime. Particular attention is paid to the configurations involved and charts of the periodicity of *χ* and *η* are constructed for a range of |**B**| values. Second, we investigate the new response functions 
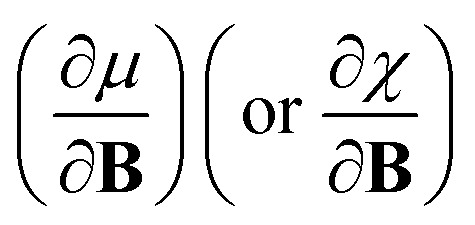
 and 
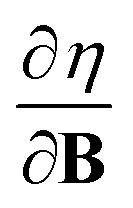
 at |**B**| = 0. These functions describe the response of *χ* and *η* to modest magnetic fields in the Coulomb regime, including those that may be generated under laboratory conditions.

In Section 2 we give the essentials of current-density-functional theory and conceptual DFT relevant to the present work and in Section 3 the computational details for the calculations are given. In Section 4, we commence with a case study on the carbon atom in Section 4.1 to highlight how configurational changes as a function of |**B**| give rise to extra complexity in studying the electronegativity and hardness as a function of the magnetic field strength. In Section 4.2, a periodic table of electronegativity and hardness is constructed and discussed for one |**B**| value (0.5 *B*_0_), the central point of the range of magnetic field strengths 0–1 *B*_0_ studied in this work. Finally in Section 4.3, the response functions 
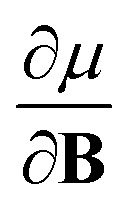
 and 
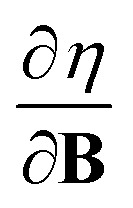
 at |**B**| = 0 together with their periodicity are addressed.

## 2 Theory

### 2.1 Current-density-functional theory

To study the descriptors *μ* and *η* in the presence of magnetic fields in the range 0–1 *B*_0_ and their response to the field at |**B**| = 0, we require the ground state energies and electronic configurations of the neutral, cation and anion species in the presence of magnetic field.

The non-relativistic electronic Hamiltonian for an *N*-electron system in a magnetic field **B** can be written as,1
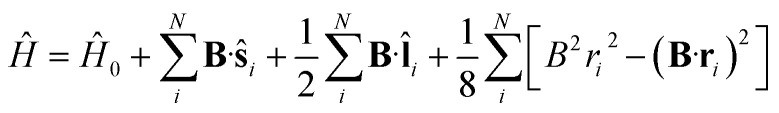
where the first term is the unperturbed zero-field electronic Hamiltonian. The linear Zeeman terms are associated with the spin (**ŝ**_*i*_) and orbital angular momentum (**l̂**_*i*_ = −*i***r**_*i*_ × ∇_*i*_) operators, describing the interaction of the electron *i* with the magnetic field **B**. These terms split the energy levels and may raise or lower the energy relative to that in the absence of a field. The remaining term, which is quadratic in **B**, is purely diamagnetic and raises the energy of the system relative to zero-field. At sufficiently high field strengths, the diamagnetic term will always dominate since it is quadratic in field strength. For atomic systems with the field oriented along the *z*-axis, the Hamiltonian of eqn (1) can be written as (see for example ref. 13),2
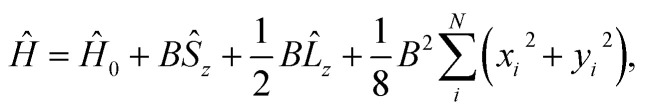
in which 
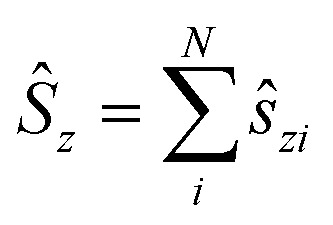
 and 
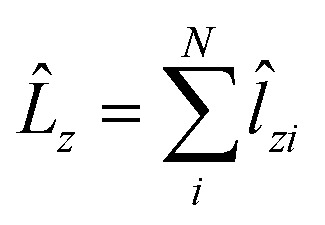
.

The range of field strengths from |**B**| = 0.0 to 1.0 *B*_0_ spans both the Coulomb and intermediate regimes. At low field (|**B**| ≪ 0.1 *B*_0_), the Coulomb interactions present in *Ĥ*_0_ are much more significant than the magnetic interactions, which may be treated perturbatively, whereas at higher fields in the intermediate regime, both interactions are of comparable strength – preventing the treatment of either by perturbative approaches. For very strong fields, typically much higher than 1.0 *B*_0_ for the atomic systems in this work, the Landau regime is entered. This regime is not considered in the present work however an interesting study of many-electron systems in this context may be found in the work by Wunner *et al.* on the series He to Si for fields extending to 5 × 10^8^ T.^48^

In the present work, we use a non-perturbative implementation of current-density-functional theory, suitable for systems in external magnetic fields of strength in the range 0.0–1.0 *B*_0_ considered in this study. Implementations of non-pertubative calculations have been developed for general atomic and molecular systems at the Hartree–Fock,^14,49^ configuration interaction,^16^ complete active space self-consistent field, Møller–Plesset, coupled-cluster and current-density-functional theory (CDFT)^50^ levels in recent years. In the presence of a magnetic field, density-functional theory (DFT) must be extended as shown by Vignale and Rasolt^51^ since the energy is no longer dependent only on the charge density *ρ* but also on the paramagnetic current density **j**_p_. As shown in ref. 52 and 53, a formulation of CDFT analogous to Lieb's convex-conjugate formulation of DFT can be constructed by re-writing the energy functional *E*(*v*, **A**) depending on the external scalar potential *v* and the vector potential **A** associated with the magnetic field in terms of the modified scalar potential 
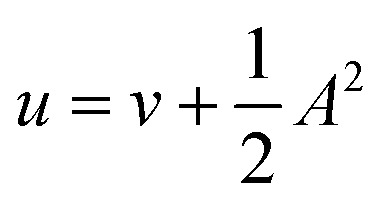
. This leads to the concave energy functional3
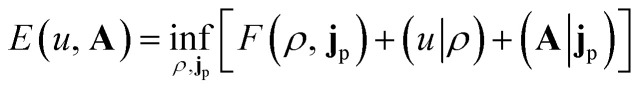
where (*u*|*ρ*) = ∫*u*(**r**)*ρ*(**r**)d**r**, (**A**|**j**_p_) = ∫**A**(**r**)·**j**_p_(**r**)d**r** and the convex-conjugate universal density functional is4
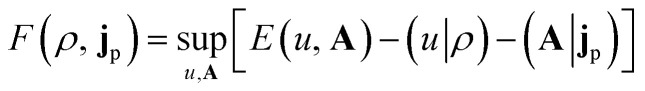
which can be identified as the Vignale–Rasolt functional.^51^

Adopting the Kohn–Sham (KS) ansatz, the functional in eqn (4) can be decomposed as

5 *F*(*ρ*, **j**_p_) = *T*_s_(*ρ*, **j**_p_) + *J*(*ρ*) + *E*_xc_(*ρ*, **j**_p_)

in which the first term is the non-interacting kinetic energy, *J*(*ρ*) the classical Coulomb electron–electron repulsion and *E*_xc_(*ρ*, **j**_p_) the exchange–correlation energy, which now depends on both *ρ* and **j**_p_.

The KS-CDFT equations take the form6
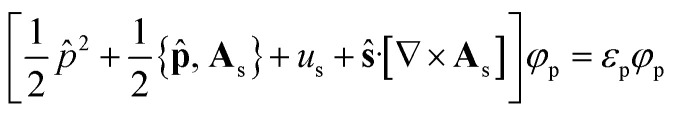
where **p̂** = −*i*∇ is the canonical momentum operator, **ŝ** is the spin operator, *ε*_p_ are the orbital energies and *φ*_p_ are the molecular orbitals. The charge and paramagnetic current densities can be expressed in terms of the molecular orbitals as7
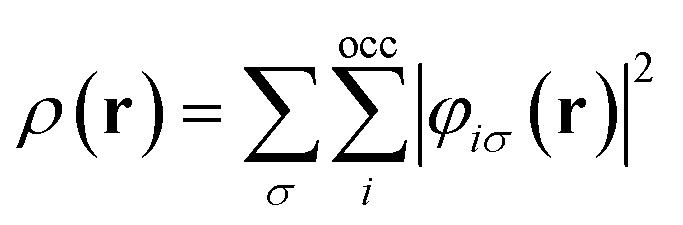
and8
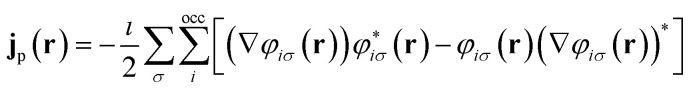
respectively, where *i* denotes occupied orbitals and *σ* their spin. The KS scalar and vector potentials are9
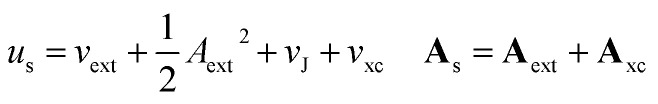
where (*v*_ext_, **A**_ext_) are the physical external potentials, *v*_J_ is the Coulomb potential, and the exchange–correlation potentials have scalar and vector components given respectively by10
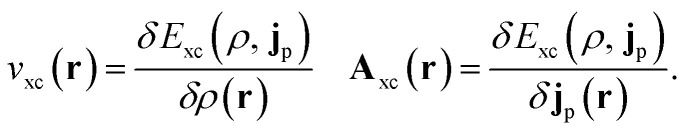


In the present work the KS-CDFT equations are implemented in an unrestricted manner.

To ensure gauge-origin independence of the calculated energies, the molecular orbitals *φ*_p_ are expanded in a set of London atomic orbitals (LAOs).^15^ These have the form11
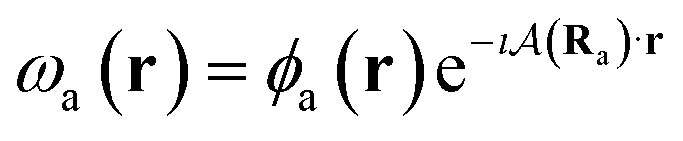
where 
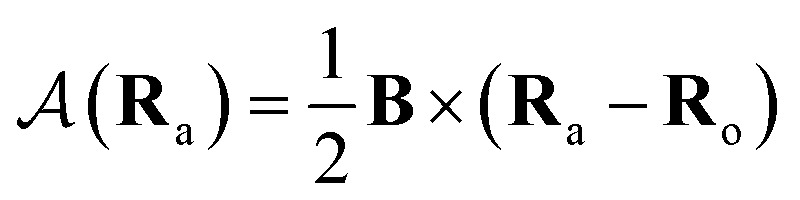
 is the vector potential at the position of the center of the LAO **R**_a_, relative to the gauge-origin **R**_o_. This defines a complex phase factor, which multiplies a standard Gaussian basis function *ϕ*_a_(**r**). We note that in the present work on atoms the gauge-origin may always be chosen to coincide with the atomic centre, reducing the LAOs to a set of complex Gaussians. However, the framework presented here is general and the quantities analysed may be calculated for atoms and molecules alike.

The remaining challenge is then to select an appropriate form for the exchange–correlation energy in practical calculations. We have previously shown that the meta-GGA level cTPSS functional^54^ provides good accuracy in the presence of strong magnetic fields.^55^ At the meta-GGA level the modified kinetic energy density12
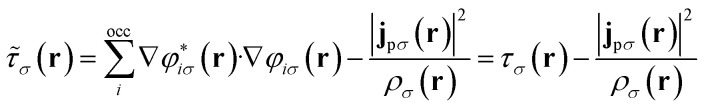
is used in place of the usual form *τ*_*σ*_(**r**) to ensure that *E*_xc_(*ρ*, **j**_p_) is independently gauge invariant, as suggested by Dobson^56^ and employed previously by Becke.^57^ Utilising this approach, a family of cTPSS functionals have been applied in strong magnetic fields^58–60^ including hybrid and range-separated hybrid variants. In the present work we will make use of the cTPSS meta-GGA and compare our results with those obtained at the Hartree–Fock level.

### 2.2 Conceptual density-functional-theory

The conceptual DFT descriptors *μ*, *χ* and *η* at a given field strength, *μ*(**B**), *χ*(**B**) and *η*(**B**) were calculated using the well-known finite difference approach^20,22^ as13
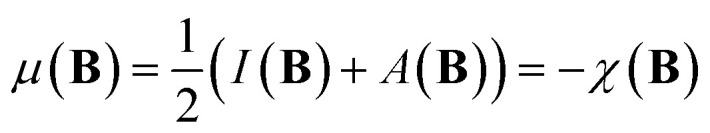
14
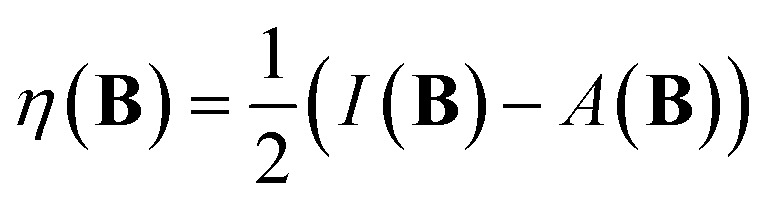
where *I*(**B**) and *A*(**B**) are the ionization energy and electron affinity at a given field strength |**B**|. It is well known that these quantities correspond to the left and right side derivatives of the energy with respect to the particle number *N*, as described by Perdew *et al.*^61^ The expressions for *μ* and *η* correspond to the averaged sum and difference of these derivatives, respectively. The factor of 1/2 present in eqn (14) is consistent with the original definition of Parr and Pearson^26^ however is often omitted from the definition of the hardness in more recent work; here we keep this factor for reasons of symmetry between the formulae for the electronegativity and hardness.

As outlined previously, the central theme of this study is the extension of the *E* = *E*(*N*, *v*) functional with an external magnetic field, leading among others to reactivity descriptors of the type 
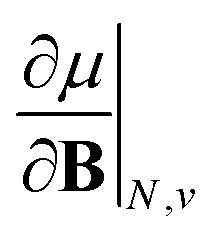
. We also note that, upon including spin in the *E* functional, a spin polarized version of conceptual DFT with functional *E* = *E*(*N*, *N*_S_, *v*, **B**) was put forward^32,33^ with *N*_S_ being the difference between the number of α and β electrons. It is tempting to see if the reactivity descriptors as evaluated in our study, *e.g.*
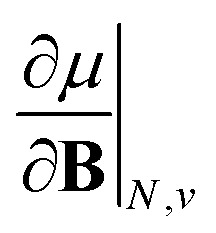
, could be considered in the context of this functional. It should however be noted here that upon adding or subtracting a complete electron, either α or β, as is the case when evaluating *I* or *A*, and seeking the lowest energy for the given value of *M*_s_, *N*_S_ is not constant: this could only occur by adding or subtracting 0.5 α and 0.5 β electrons, which would not lead to the ground state configuration. In summary, the condition of constant *N*_S_ in the partial derivatives with respect to **B**, in the context of the *E* = *E*(*N*, *N*_S_, *v*, **B**) functional, is not compatible with obtaining the ground state energy.

## 3 Computational details

For the Hartree–Fock calculations in Subsection 4.1, which allow for comparison with literature data, a q-aug-cc-pVQZ basis was used.^62–67^ All CDFT calculations were carried out using the cTPSS exchange–correlation functional,^54,68^ a modified version of the TPSS functional^69^ belonging to the meta GGA class of functionals, the prefix c indicating that a modified version of the functional was used including the current density (through the modified form of the kinetic energy density given in eqn (12)), suitable for calculations in the presence of strong magnetic fields.^55^ This functional has been shown to perform well relative to high-level *ab initio* calculations in strong magnetic fields in ref. 55. Preliminary studies for a subset of atomic systems also indicated that the use of a hybrid or range-separated hybrid variants of this functional (see ref. 58) does not significantly alter the results in the present work. For these calculations, the d-aug-cc-pV5Z Gaussian basis set was employed.^62–67^ As the aim of our study was to cover the main group elements up to and including the fourth row elements, K and Ca had to be excluded as this basis was not available for these elements.The magnetic field was oriented along the *z*-axis in all calculations.

The calculation of 
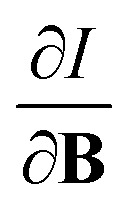
 and 
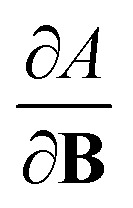
 derivatives was done in a finite field approach by straight line fitting of the |**B**| = 0, 0.0001, 0.001, 0.01, 0.02 and 0.04 *B*_0_ results unless a change in electronic configuration was observed, in which case only those energies up to the field strength at which this occurs were used. These values are combined into 
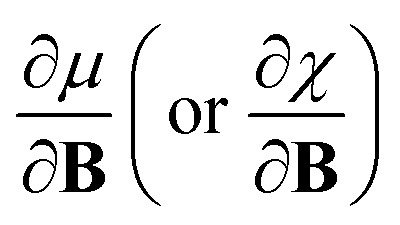
 and 
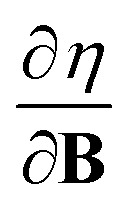
 values using the derivative forms of eqn (13) and (14) respectively. This approach was not always straightforward since, for some species, a discontinuity appears in the *E vs.* |**B**| curve approaching zero field, with the energy at |**B**| = 0 not being that of the ground state. This is a manifestation of the multiplet problem associated with single–determinant methods such as HF and KS DFT, in which configurations which should be degenerate such as those in the ^3^P zero-field ground state of the carbon atom do not have the same energy with such methods.^56,70^ However, in the presence of a magnetic field this degeneracy is lifted and also the different components of the multiplet exhibit a different variation in energy with respect to field strength. Therefore, to ensure the *E vs.* |**B**| curve is continuous approaching zero field, calculations were first undertaken at a higher field strength (typically 0.1 *B*_0_) and the converged orbitals used as the initial guess for the lower and zero-field calculations. In this way, a selected electronic configuration could be traced back to zero field. In each case we selected the component of the multiplet that becomes the ground state for very low (but non-zero) magnetic fields as the configuration to study.

For the calculations at higher field strengths, the region |**B**| in the range 0.1 to 1 *B*_0_ was scanned with intervals of 0.1 *B*_0_. For each |**B**| value, a range of *M*_s_ values of the neutral atom was considered. For each *M*_s_ value, the lowest energy and associated configuration was selected (since sometimes more than one configuration corresponds to a given *M*_s_). Comparison of the lowest energy values for each *M*_s_ then yields the ground state energy and corresponding configuration at a given |**B**| value. A similar procedure was followed for the cation and the anion, allowing the calculation of *I*(**B**) and *A*(**B**) and, from these, *μ*(**B**) and *η*(**B**) (eqn (13) and (14)). This procedure is exemplified in Subsection 4.1 for the case of the carbon atom. Since our calculations only specify *M*_s_ it is possible that the self-consistent field calculations could converge to a solution that is not the ground state. To mitigate against this, continuity of the solutions as a function of |**B**| was carefully examined, both in terms of the energy and the nature of the orbitals involved. In practice, it was found that such issues were only problematic in a few cases where configurations were nearly degenerate close to |**B**| = 0 and lower energy solutions could be readily obtained by using orbitals from higher fields and an initial guess.

## 4 Results and discussion

### 4.1 Electronegativity and hardness of the C atom

As described previously, to compute the electronegativity and hardness values at a given |**B**|, according to eqn (13) and (14) respectively, the basic ingredients are the ionization energy and the electron affinity, which should be calculated at that value of |**B**|. As the electronic configurations for the neutral system, anion and cation may (and in most cases will) differ from their zero-field counterparts, the search for an optimal configuration at a given |**B**| for the neutral, the cationic and anionic system should be conducted at that |**B**| value.

In order to illustrate this complexity and as a proof of concept, this analysis is undertaken for the carbon atom, for which the energy and associated configurations were scanned from |**B**| = 0 to 1.0 *B*_0_ as described in Section 3 for the neutral system, the cation and the anion. To the best of our knowledge this is the most extensive exercise of this type since the pioneering studies by Schmelcher on the neutral system,^47^ permitting a direct comparison for the neutral C atom, and the more intricate combined studies on the neutral atom/cation combinations for Li, Be, B by the same group.^8–10,12^

Table 1 shows the evolution of the ground state energy and configuration of the carbon atom, its cation and anion where, for each system, a change in configuration is indicated by a change in text color.

**Table 1:** Evolution of the ground state energies of C, C^+^ and C^−^ and their respective electronic configurations as a function of the magnetic field strength between 0 and 1.0 *B*_0_. Changes in configuration are indicated by changes in color. Singly occupied orbitals are always carrying a β electron (arbitrarily at |**B**| = 0). All values are calculated at the HF level with the q-aug-cc-pVQZ basis

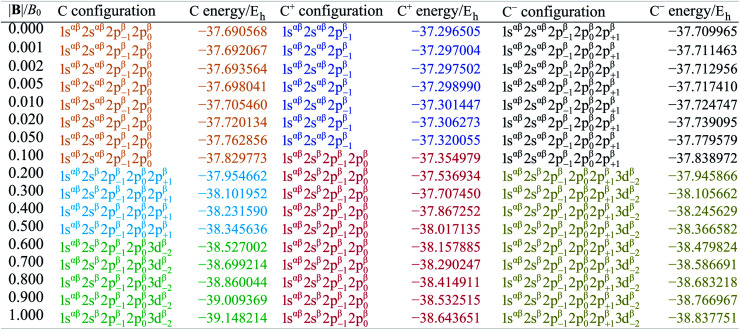

For the neutral C atom at zero-field, the lowest energy component of the ^3^P ground-state is that with the 2p_0_ and 2p_−1_ orbitals singly-occupied by electrons of the same spin, here taken to be β. This configuration remains the ground-state in a very small field, since it maximises the number of β electrons and minimises the *M*_L_ value thus has the greatest decrease in energy with increasing field strength. At |**B**| = 0.2 *B*_0_ the two 2s electrons decouple, giving rise to two extra unpaired β electrons with concomitant stabilization due to the spin-Zeeman effect, despite an electron now occupying the higher energy 2p_+1_ orbital (blue region). The *M*_s_ value thereby jumps from −1 to −2. At higher field, the configuration with an occupied 3d_−2_ orbital eventually becomes the ground state, since the energy of the 3d_−2_ orbital decreases with field strength due to the orbital paramagnetic effect and eventually falls below that with the 2p_+1_ orbital, resulting in the ground-state configuration changing from …2p_−1_2p_0_2p_+1_ to a 2p_−1_2p_0_3d_−2_ (green region) but the value of *M*_s_ remaining −2. This behavior is in line with that reported by Schmelcher.^47^ Values are not identical due to differences in computational approach: whereas a numerical HF approach was followed by Schmelcher, the present calculations were carried out using a finite basis set expansion. The most important difference is that, whereas the *M*_s_ = −1 to −2 transition occurs at a similar field strength (0.19 *B*_0_*vs.* 0.20 *B*_0_), the change between the two *M*_s_ = −2 configurations occurs at a lower field strength in Schmelcher's study compared to this work (0.49 *B*_0_*vs.* 0.60 *B*_0_). It should be noted that a smaller size of interval in |**B**| could be used to refine the field strength at which these transitions occur in the present work. Important is that the energy differences are on average 0.0006 *E*_h_ for field strengths below |**B**| = 0.05 *B*_0_ and 0.006 *E*_h_ for field strengths between 0.5 and 1.0 *B*_0_.

Overall, the comparison with Schmelcher's work suggests that the computational methods employed here are reliable and can be applied to analyse the cationic and anionic states of carbon. At low magnetic field strengths, the 1s^2^2s^2^2p_−1_ configuration of the cation is lowest in energy, having values of *M*_L_ = −1 and 
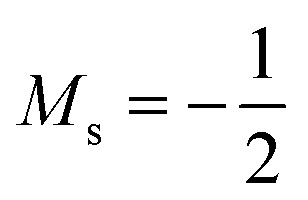
, indicated by purple text in Table 1. At a magnetic field of |**B**| = 0.10 *B*_0_, 1s^2^2s2p_−1_2p_0_ becomes the ground-state electronic configuration (red text in Table 1) as a result of the unpairing of the 2s electrons, with a resulting change in *M*_s_ to 
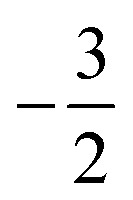
.

As for the cation, only two configurations are observed for the anion over this range of field strengths: the 1s^2^2s^2^2p_−1_2p_0_2p_+1_ configuration from |**B**| = 0.0 to 0.1 *B*_0_ with 
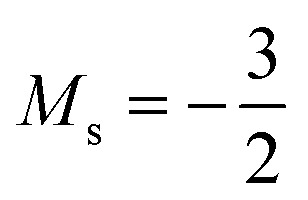
 and, by the unpairing the 2s electrons at |**B**| > 0.1 *B*_0_, the 1s^2^2s2p_−1_2p_0_2p_+1_3d_−2_ with 
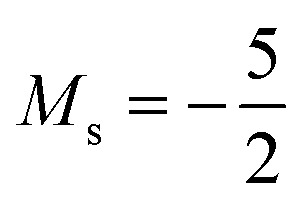
. It is interesting to note that the field strengths at which the ground state configurations of the carbon anion, cation and atom change do not generally coincide, indicating that care should be taken when interpreting variations in the ionisation energy and election affinity as a function of the magnetic field strength. Finally, it can be seen that from |**B**| = 0.6 to 1.0 *B*_0_, the energy of the anion is higher than that of the atom, resulting in a negative electron affinity; for a more detailed discussion see Subsections 4.2 and 4.3.

The behaviour of *I* and *A* as a function of magnetic field strength are summarised in Table 2. Different colours are again used to indicate the regions in |**B**| where either of the two species involved in the calculation of these quantities changes its ground-state electronic configuration and through which *I* and/or *A* are expected to vary smoothly with |**B**|. As a result of this analysis, the ionisation energy can be split into four ‘segments’: from |**B**| = 0.0–0.05 *B*_0_, 0.1 *B*_0_, 0.2–0.5 *B*_0_ and 0.6–1.0 *B*_0_, indicated in Table 2. Though in each segment the electron is removed from a different orbital in the atom to form the cation (2p_0_, 2s, 2p_+1_, 3d_−2_ respectively), remarkably the same cation is formed in segments 2, 3 and 4, but each time from a different neutral system configuration.

**Table 2:** Evolution of ionization energy, electron affinity, electronegativity and hardness as a function of field strength |**B|**. Different segments (see text) are indicated by different colors. Orbitals from which an electron is taken (*I*) or to which an electron is added (*A*) are indicated

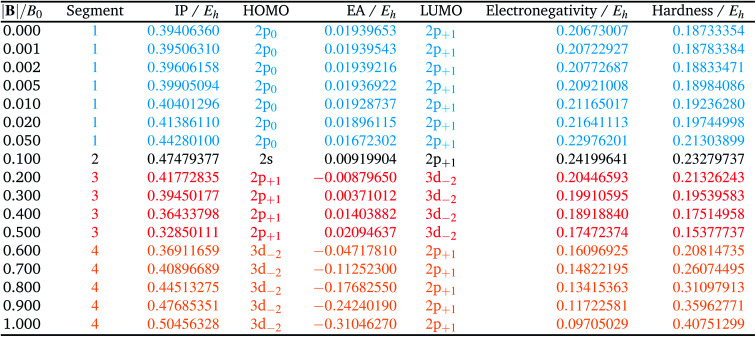

**Fig. 1:**
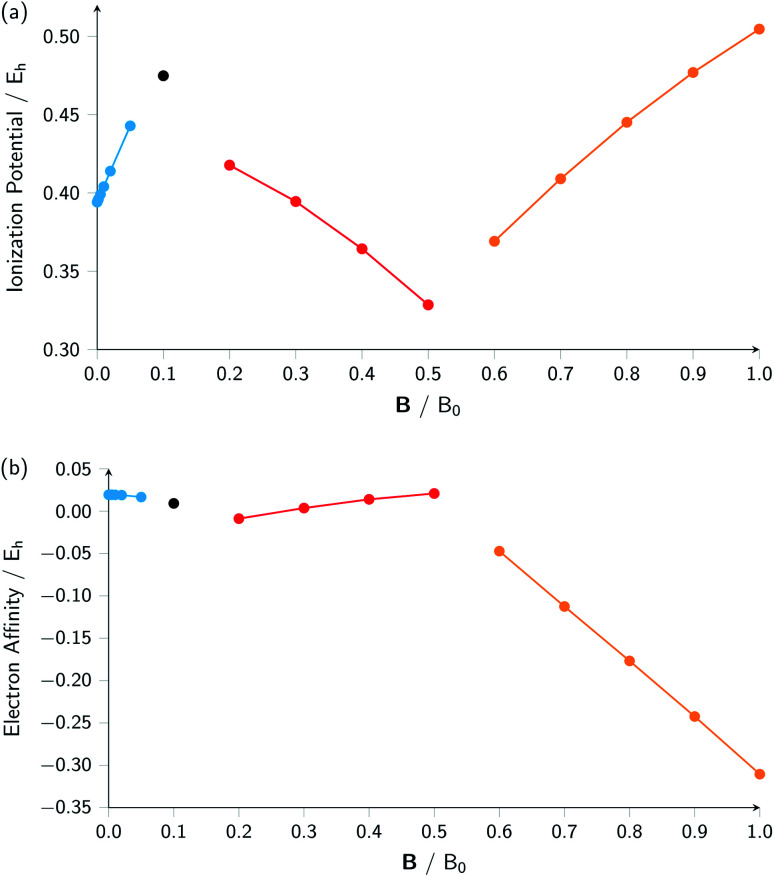
Ionization energy (a) and electron affinity (b) *versus* field strength |**B**| for the carbon atom (all values in atomic units). The different segments correspond to the different colors in Table 2.

A similar observation can be made for the electron affinity, for which three regions are discerned where the attached electron occupies the 2p_+1_, 3d_−2_ and then 2p_+1_ orbitals, but leading in the last two cases to the same configuration; in the first case from |**B**| = 0.2–0.5 *B*_0_ the LUMO of the atom is the 3d_−2_ orbital which becomes occupied in the formation of the anion, whilst in the second case |**B**| > 0.5 the 3d_−2_ orbital is the HOMO of the atom and its LUMO is the 2p_+1_ orbital which becomes occupied on formation of the anion. Again, considering the electron affinity as a function of magnetic field strength, discontinuities between the three or four segments can be discerned, which can be seen more clearly in Fig. 1 where we depict plots of *I* and *A* as a function of the field strength.

Fig. 1 clearly shows how different the behavior of both *I* and *A* can be in different regions of |**B**|: differences in slope (both in magnitude but even in sign) show up, making a discussion on the behavior for the two quantities from |**B**| = 0.0 to 1.0 *B*_0_ perhaps unexpectedly complicated from a more chemical point of view. Their non-uniform behavior indeed hampers typical chemical intuition based thinking/reasoning: when a system is perturbed, a given quantity is either unchanged or it increases or decreases with, in the last two cases, a greater effect when the perturbation is larger. The value of response functions at |**B**| = 0.0 thereby clearly emerges. This type of behavior was also found by Schmelcher and coworkers for the ionization energy of Li, Be and B, where for Li and Be the same piecewise behaviour of the *I*(**B**) curve was found in the range from |**B**| = 0 to the fully decoupled states at very large field strength^8,10^ and where also for B a difference in the piecewise behaviour of the *E vs.* |**B**| curve between the neutral system and the cation was highlighted in that same range.^9^ The analogous behavior of the electron affinity is not unexpected in view of the fundamental role which is played by changes in configurations of any atomic system as a function of an external magnetic field.

**Fig. 2:**
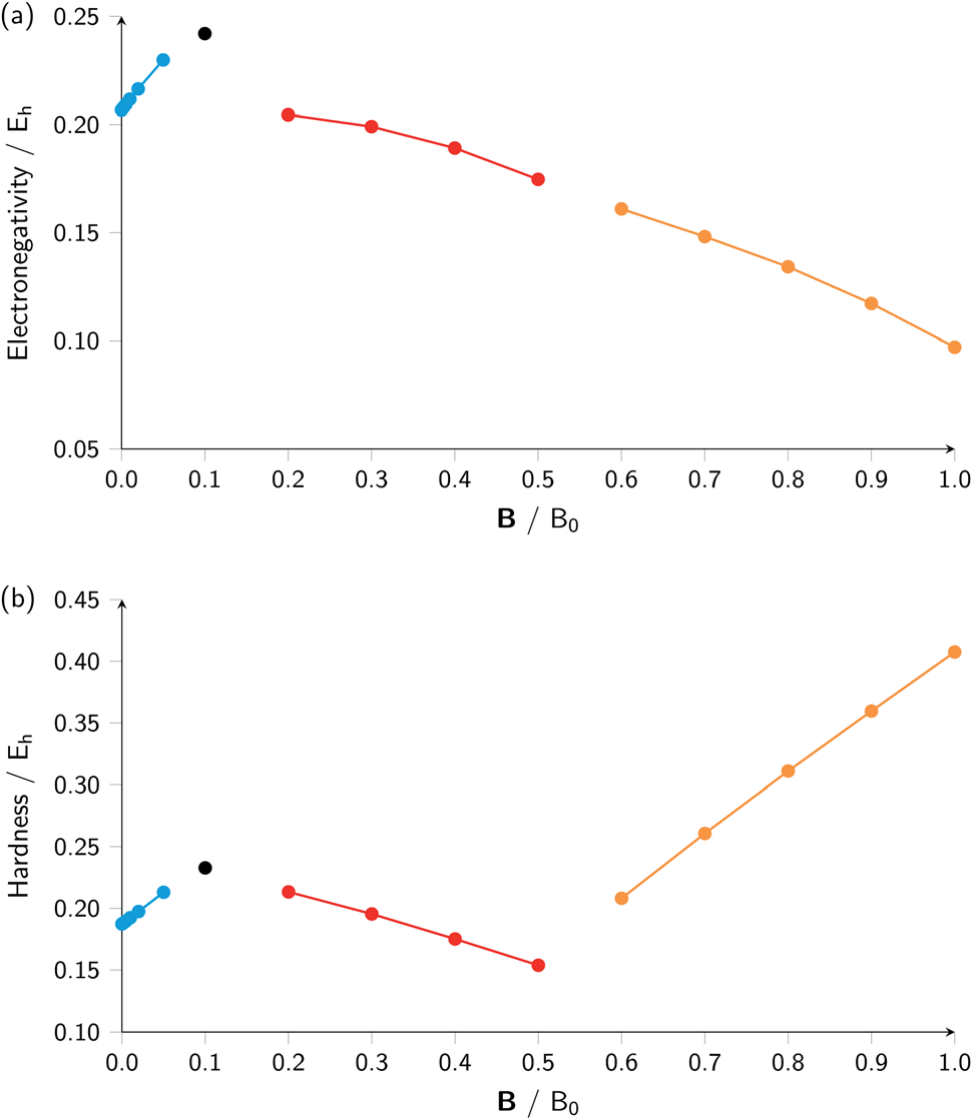
Electronegativity (a) and hardness (b) *versus* field strength for carbon (all values in a.u.). Different segments correspond to the color codes in Table 2.

Combining ionization energy and electron affinity into Mulliken's electronegativity in eqn (13) and Pearson's hardness expression in eqn (14) leads to further complications since then two quantities with their own, often different “segments” in their |**B**| variation are to be combined, possibly leading to a further segmentation in the |**B**| variation of the electronegativity and/or hardness. In the case of carbon, by chance, there is no such further complication since the segmentation pattern of *I* and *A* in |**B**| are identical, leading again to four segments for both the electronegativity and the hardness (Table 2 and Fig. 2).

Concentrating initially on the electronegativity, a first regime is observed in segment one where *χ* increases as a function of |**B**| quasi linearly (from an initial value of 0.207 to 0.230 *E*_h_) with a slope of 0.46. The second regime is the single point at a higher value (0.242 *E*_h_), followed by a third regime with an almost linearly decreasing electronegativity between |**B**| = 0.2 and 0.5 *B*_0_ with a slope of −0.10. The final regime, from |**B**| = 0.6 to 1.0 *B*_0_, also shows a decreasing electronegativity, again nearly linear, but with an even more pronounced decrease in *χ* (slope value −0.16). For the hardness a similar structure in the plot may be expected, and Fig. 2 indeed shows again four regions corresponding with the segments in Table 2 with different slopes for the linear variation of *η* with respect to |**B**| in segments one, three and four.

It can be seen from Fig. 2 that the behaviour of electronegativity and hardness as a function of |**B**| for different atoms is extremely subtle such that predictions are more difficult to make: from the knowledge of the behavior of an atom of a given element, it is far from clear what the behavior of an atom from a different element in a magnetic field will be. This issue will nevertheless be addressed in Subsection 4.2 where we try to discern patterns throughout the periodic table at a given |**B**| and in Subsection 4.3 where response functions at zero field are discussed.

As stated previously, extensive calculations (encompassing all main group elements from H to Ar, except for K and Ca) were carried out with CDFT using the cTPSS exchange–correlation functional on account of its higher accuracy compared to Hartree–Fock (HF), although the results are qualitatively similar when considering the transitions in *M*_s_. In the case of the carbon atom, the transition from *M*_s_ = −1 to −2 occurs at 0.3 *B*_0_ with cTPSS compared to 0.2 *B*_0_ with HF, whilst for its cation the transition from 
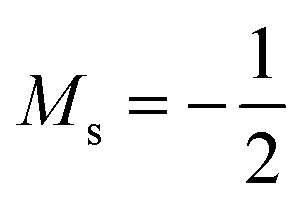
 to 
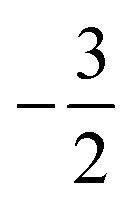
 occurs at 0.1 *B*_0_ in both cases and for the anion the transition from 
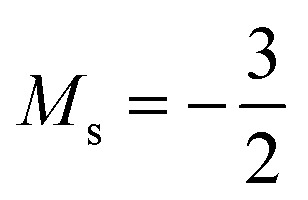
 to 
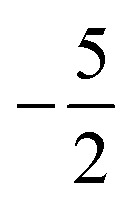
 occurs at 0.2 *B*_0_ with cTPSS compared to 0.1 *B*_0_ with HF.

### 4.2 Towards a periodic table of the electronegativity and hardness at a given |**B**| (0.5 *B*_0_)

**Fig. 3:**
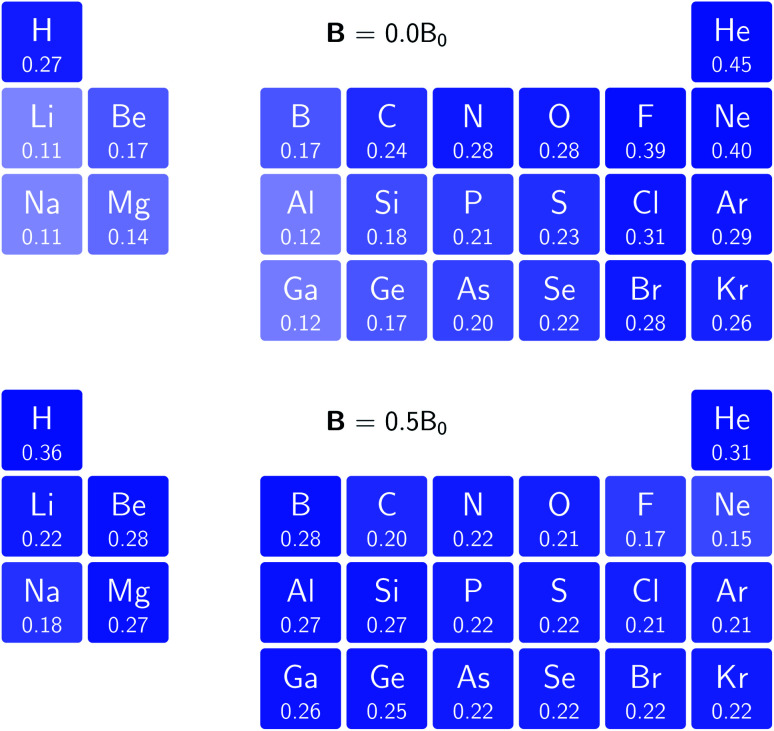
Schematic representation of the electronegativity values at |**B**| = 0.0 (upper) and |**B**| = 0.5 *B*_0_ (lower). Darker colors indicate higher electronegativity.

**Fig. 4:**
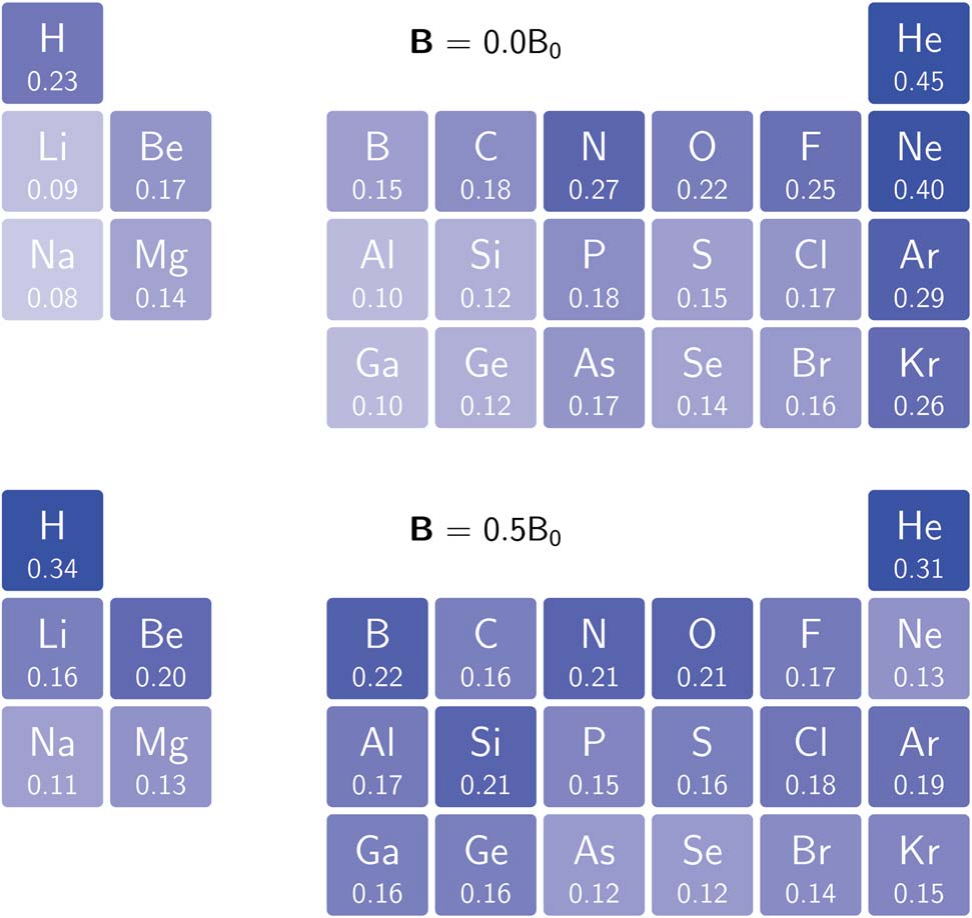
Schematic representation of the hardness values at |**B**| = 0.0 (upper) and |**B**| = 0.5 *B*_0_ (lower). Darker colors indicate greater hardness.

In Fig. 3 and 4 we depict the electronegativity and hardness values respectively for the main group elements up to Kr (except K and Ca), both at |**B**| = 0.0 and 0.5 *B*_0_, this value having been chosen as the halfway point in the range of field strengths considered here, to give an overview of the periodic table of these properties in strong magnetic fields compared to at zero field. In these CDFT studies, as mentioned in Subsection 4.1, special care was taken in the case of negative electron affinity values for all elements and all field strengths. In case of an unstable anion, the corresponding electron affinity was set to zero, as is often done in conceptual DFT approaches. Please note that this strategy is different from that in the previous part of the paper where negative electron affinities were kept as such. The data in the ESI† also adopts this strategy. Both strategies have been advocated in the literature.^71^

Fig. 3 retrieves for |**B**| = 0 the well known trends of increasing electronegativity from left to right (neglecting the noble gases for the moment) and from bottom to top in the periodic table. The lowest value is found at the bottom left (Na) and the highest value at the top right (F), as expected and in line with literature data.^20^ The high electronegativity value for the noble gases, not reported in all tables and for example absent in the Pauling scale, arises due to their extremely high ionization energies (see the discussion in ref. 72 by some of the present authors) and negative electron affinities, which are taken to be zero as described above.

Generally positive values of *A* result in an increased value *χ*, but since the ratio of electron affinity (when positive) and ionization energy is typically only of the order of 1/8, the final result is that the very high ionization energies of the noble gases is sufficient to yield electronegativity values of the order of their halogen congeners, even resulting in He having the highest electronegativity. In the more detailed study in ref. 72, it was concluded that the combination of their high electronegativity with their extreme hardness determines the chemistry of the noble gases.

Considering now the case at |**B**| = 0.5 *B*_0_, it first should be noted that all anions at this |**B**| value were found to be stable since the addition of a β electron further stabilises the system due to the spin-Zeeman effect. This fundamental difference with the |**B**| = 0 case has only three exceptions: He with a negative electron affinity of −0.009 *E*_h_ and, remarkably O and F with electron affinities of −0.0007 *E*_h_ and −0.0175 *E*_h_ respectively. For He and O, the electron affinities become positive for |**B**| = 0.5 *B*_0_ to 1.0 *B*_0_ whereas for F the electron affinity is positive for |**B**| = 0.5 *B*_0_ to 0.7 *B*_0_, turning negative for |**B**| = 0.8 *B*_0_ to 1.0 *B*_0_. At higher fields intricacies with the configurations of neutral and anionic system result in an unexpected behavior. Again, in cases were *A* was negative *χ* was evaluated as *I*/2.

When considering the overall trends in electronegativity, it can be seen that the pattern at |**B**| = 0.5 *B*_0_ is significantly different to that at zero field; the first four columns except for carbon strongly increase their electronegativity, the elements of columns 5 and 6 show a slight decrease, whereas the halogens and noble gases exhibit large decreases. This will be compared to the behaviour of the initial response of the electronegativity at zero field in Subsection 4.3.

Overall the impression is that the electronegativity values show a tendency to be compressed in a smaller range in a strong magnetic field. The result is that, in these conditions, the left hand side of the periodic table generally shows higher electronegativity values than the right hand side, leading to fundamental changes in chemistry (*e.g.* the polarity of bonds).

**Fig. 5:**
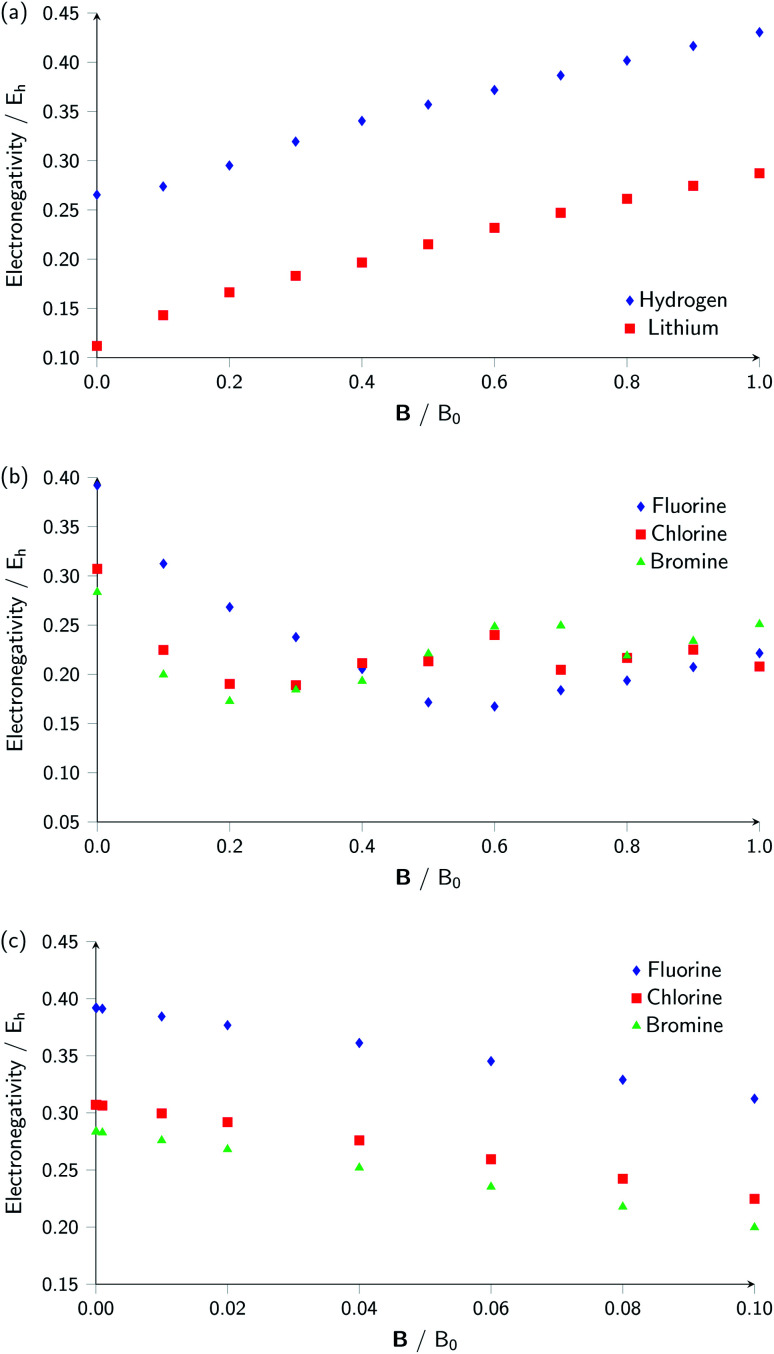
(a) Electronegativity of hydrogen and lithium in the range |**B**| = 0 to 1.0 *B*_0_, (b) electronegativity of the halogens (F, Cl, Br) in the range |**B**| = 0 to 1.0 *B*_0_ and (c) electronegativity of the halogens (F, Cl, Br) in the range |**B**| = 0 to 0.1 *B*_0_.

Whilst it can be difficult to find trends in behaviour across a period due to the many discontinuities arising from changes in ground-state configuration of the elements at different field strengths, it can be interesting to compare the behaviour of elements in the same group with respect to magnetic field strength since the pattern of changes in ground-state configuration may be similar. This analysis is presented in Fig. 5 for certain columns of the periodic table. In Fig. 5(a) the variation in electronegativity with magnetic field for H and Li are shown; they both have a roughly linear variation with field strength, most likely because both atoms have 
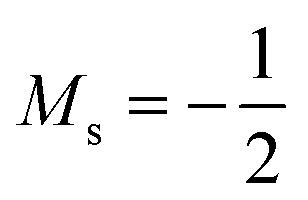
 and cations have *M*_s_ = 0 from |**B**| = 0.0 to 1.0 *B*_0_, whilst both anions have *M*_s_ = −1 from at least |**B**| = 0.1 *B*_0_ to 1.0 *B*_0_.

The situation is different for the halogens, where the well-known zero-field situation of *χ*_F_ > *χ*_Cl_ > *χ*_Br_ evolves in a different way between F and its two heavier congeners, as can be seen in Fig. 5(b). Concentrating, for the sake of simplicity on the configuration of the neutral atoms, F only undergoes one transition from 
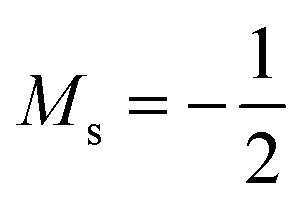
 to 
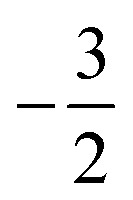
 at |**B**| = 0.6 *B*_0_ whereas Cl and Br undergo three changes from 
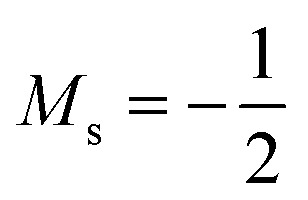
 to 
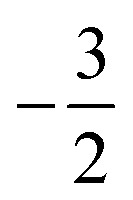
 then to 
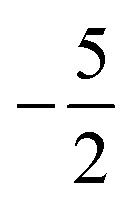
 and 
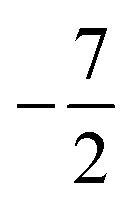
 at |**B**| = 0.3 *B*_0_, 0.5 *B*_0_ and 0.7 *B*_0_ respectively. This is reflected in Fig. 5(b), where it can be seen that the slope of electronegativity with respect to magnetic field changes sign only once for F whereas it changes at least three times for Cl and Br. This difference leads to the remarkable effect that in the |**B**| = 0.4–0.6 *B*_0_ region *χ*_F_ is still decreasing whereas *χ*_Cl_ and *χ*_Br_ increase and lie well above *χ*_F_. At |**B**| > 0.6 *B*_0_, *χ*_F_ steadily increases whereas Cl and Br show an oscillatory behavior due to changes in the ground-state configuration, resulting in a F, Cl, Br sequence which changes several times.

Fig. 5(c) shows the variation of electronegativity with field strength for the halogens from |**B**| = 0.0–0.1 *B*_0_; in this region, the three curves behave in a similar way and no changes in *M*_s_ occur in that region, indicating furthermore that taking response functions at |**B**| = 0 would be useful to investigate.

The hardness values in Fig. 4 at zero field exhibit the well-known behavior of alternating values along a period^10,20,73^ with maxima for the second, fifth and eighth groups with unoccupied, half-filled and fully occupied 2p shells respectively.^72^ In each column a uniform decrease is observed, corresponding with increasing softness/polarizability. At |**B**| = 0.5 *B*_0_ nearly all atoms up to the fifth group show an increase in hardness relative to zero field, but at this and subsequent groups the hardness is decreased relative to zero field (with some minor exceptions), being most pronounced for fluorine and especially the noble gases. The result is that values at the left and at the right of the periodic table become closer to each other and, just as in the case of the electronegativity, an overall compression of the hardness range is observed. Note however that, with exception of Si and Ne, in a given column the tendency of decreasing hardness moving down in the periodic table is preserved.

Combining the observations from the changes in electronegativity and hardness, relevant changes in the chemistry of main group elements compared to zero field could be predicted, for example using Huheey's expressions for bond polarity and bond energy based on the electronegativity equalization principle.^22,74^ Both quantities indeed reduce to a difference in electronegativity (squared in the case of bond energy) modulated by a sum of hardness values.

### 4.3 Response functions at |**B**| = 0

**Fig. 6:**
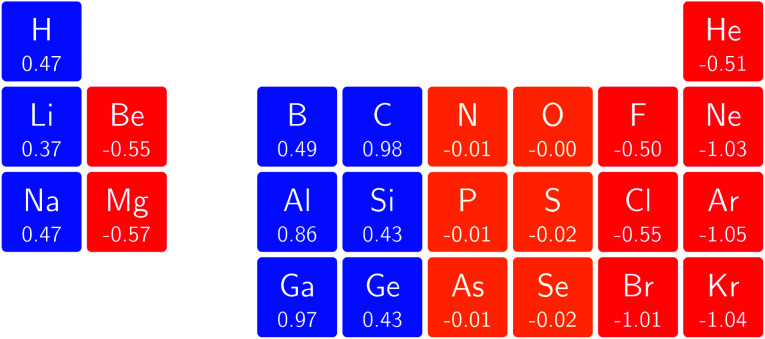
Initial responses of the ionization energy in a magnetic field as a periodic table representation showing both the numerical values and categorising them with a color code in which blue indicates a positive derivative, red a negative derivative and yellow a derivative that is zero or close to zero.

One way in which the magnetic field-dependence of these conceptual DFT quantities can be examined without the complications of the changes in ground-state configuration that occur as the magnetic field strength increases is by considering the derivative of these quantities with respect to field strength, evaluated at zero field. These response quantities should lend themselves best to an overall comparison between the behavior of the main group elements. In Fig. 6 we depict the initial response of the ionization energy, with a periodic table representation showing both the numerical values and categorising them with a color code in which blue indicates a positive derivative, red a negative derivative and yellow a derivative that is zero or close to zero.

From these response properties, a periodicity can be discerned which is different to that for the zero field electronegativity. In this case, the derivative is positive for the first column of the s-block elements but negative for the second column. A similar pattern emerges for the p-block elements: starting from positive values in columns 3 and 4, they pass to nearly zero in columns 5 and 6 and end with highly negative values for the halogens and the noble gases. Within a given column where no changes in ground-state configuration are expected at the very low field values used to obtain the derivative, neither for the neutral system nor for the cation, the values turn out to be of the same order of magnitude, be it with a few exceptions, without displaying a particular pattern.

**Fig. 7:**
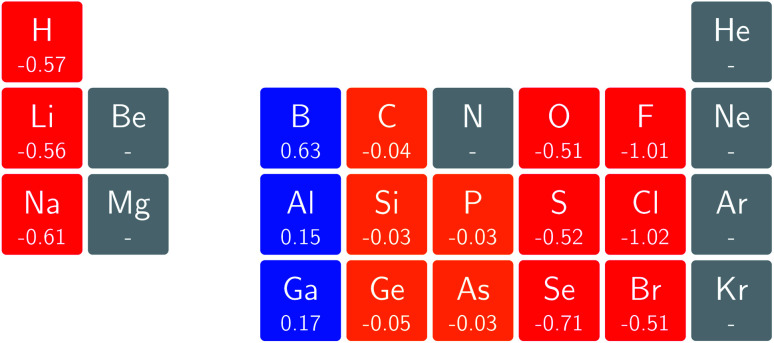
Initial responses of the electron affinity in a magnetic field as a periodic table representation showing both the numerical values and categorising them with a color code in which blue indicates a positive derivative, red a negative derivative, yellow a derivative that is zero or close to zero and grey those cases where electron affinity was negative and its derivative not computed (see text).

For the electron affinity, a similar presentation is given in Fig. 7 but in which cases with negative *A* values at zero field were eliminated. Again, a periodicity can be observed in which the derivatives within a given column have the same sign and are of a similar order of magnitude; the derivative is positive in the first column of the p-block but decreases to near zero in the second and third columns, becoming increasingly negative in the fifth and sixth columns of the p-block. In the halogen group, bromine substantially deviates from chlorine and fluorine; this apparent anomalous behavior was investigated in more detail and can be ascribed to the fact that the 5p orbitals are sufficiently low in energy that even at very low fields of the order of 0.0001 *B*_0_ a change in ground-state configuration occurs with the 5p_−1_ orbital becoming occupied.

When evaluating the response of the electronegativity to the magnetic field at zero field, these derivatives were calculated as the average of the derivatives of ionization energy and electron affinity, where in case of negative *A* values (indicated in Fig. 7) only the ionization energy contribution was taken into account.

**Fig. 8:**
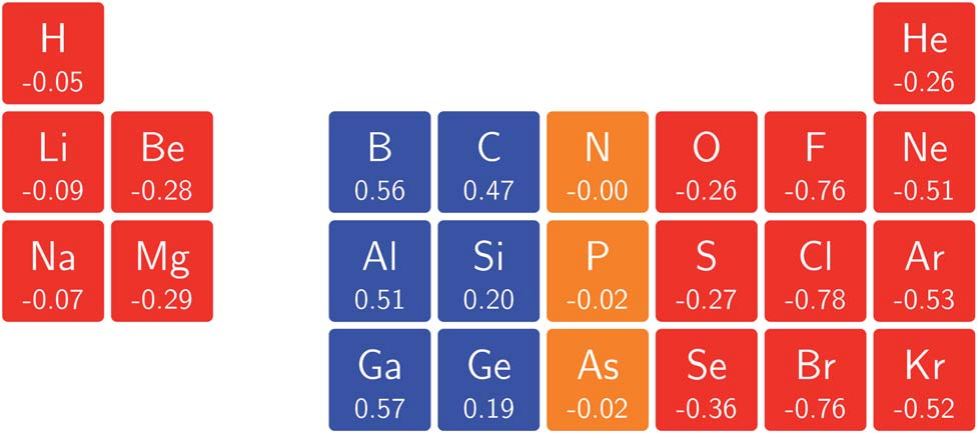
Initial responses of the electronegativity in a magnetic field as a periodic table representation showing both the numerical values and categorising them with a color code in which blue indicates a positive derivative, red a negative derivative and yellow a derivative that is zero or close to zero.

**Fig. 9:**
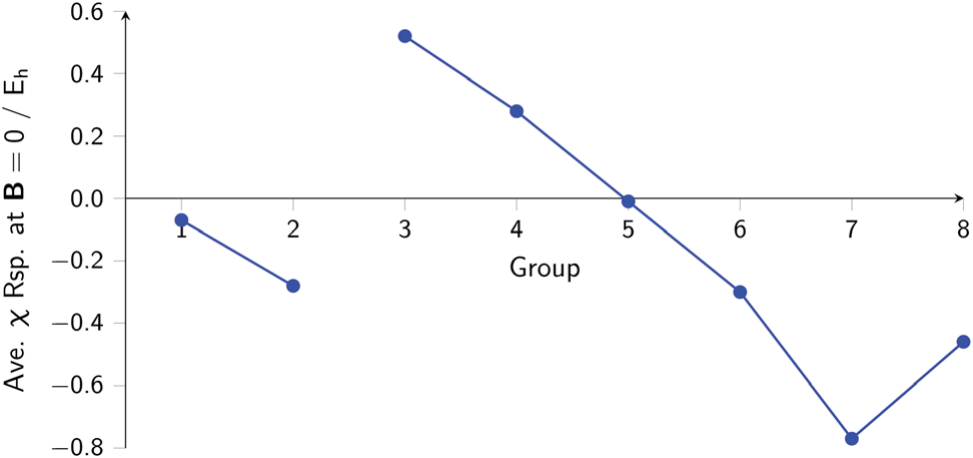
Evolution of the average electronegativity response at |**B**| = 0 for a given column in the periodic table for the main group elements.

The overall picture in Fig. 8 looks similar to that for the ionization energy in Fig. 6, the main differences being that the derivatives for the first column are slightly negative here due to the strongly negative electron affinity derivatives (the decrease in *χ* at very low |**B**| for H and Li, not visible in Fig. 5, can be seen in Tables I and III respectively of the ESI†), whilst the derivatives in the sixth column are significantly negative rather than near zero as in the case for the ionisation energy derivatives, again due to strongly negative electron affinity derivatives. For the p-block, the overall picture is similar to that for the response of *I* and *A*, with large positive values in the first column decreasing to near zero in the third column and becoming large and negative in the fifth column. In summary, the electronegativities of the elements at either end of the p-block are most sensitive to perturbation by an external magnetic field, leading to a large increase in electronegativity for elements on the left side of this block and a large decrease in electronegativity for elements on the right side. Since there is much less variation between values within a column, the average value of the response of the electronegativity to the magnetic field is evaluated for each column and presented in Fig. 9.

Although this analysis is not directly analogous to the comparison of *χ* at |**B**| = 0 and 0.5 *B*_0_ seen in Fig. 3 (due to the changes in ground-state configuration that occur over this range of field strengths), some similarity in trends can be observed for the p-block elements. The increase in *χ* in Fig. 3 for the third and fourth groups aligns with the positive derivative for *χ* with respect to field strength seen in Fig. 8, little change in *χ* for the fifth group in Fig. 3 corresponds to a near zero derivative and a decrease in *χ* for the last three groups in Fig. 3 corresponding to a negative derivative of *χ* with respect to field strength seen in Fig. 8.

Considering finally the response of hardness to an external magnetic field, it would not necessarily be expected to show the same pattern as that for the electronegativity. This is because, whilst values of *A* are generally much smaller than values of *I* for a given element resulting in a relatively small difference between *χ* and *η*, this is not necessarily the case for the derivatives of *I* and *A* with respect to the magnetic field. The magnitude of the response of *I* and *A* with respect to the field can be of the same order of magnitude; as such, the response of *χ* (calculated from the sum of the responses of *I* and *A*) can be very different to that of *η* (calculated from the difference of the responses of *I* and *A*).

**Fig. 10:**
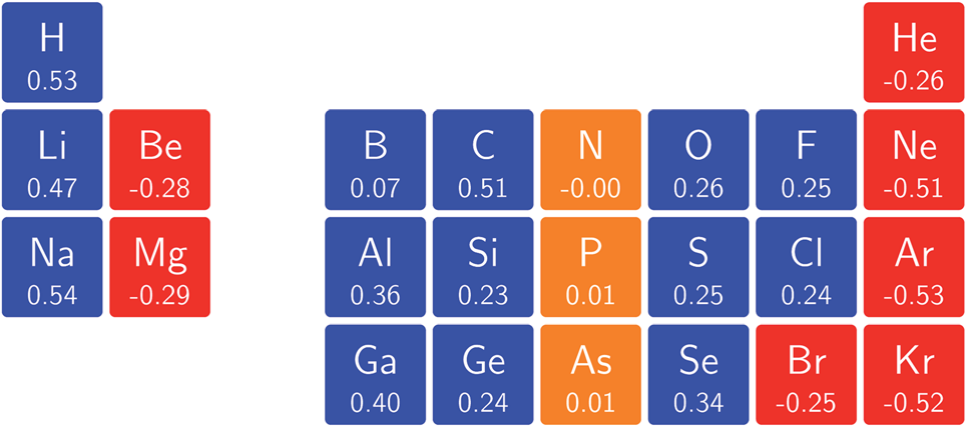
Initial responses of the hardness in a magnetic field as a periodic table representation showing both the numerical values and categorising them with a color code in which blue indicates a positive derivative, red a negative derivative and yellow a derivative that is zero or close to zero.

Fig. 10 shows that, for elements with a fully occupied valence sub-shell the initial response of the hardness with respect to the field is negative, for elements with a half-filled valence sub-shell the initial response of the hardness is near-zero and for the other elements, with partially occupied valence sub-shells, the initial response of the hardness is positive (with the exception of Br). Elements in the same column thus show a similar behaviour, although the magnitude of the response does not always follow a clear trend within a group. As for the electronegativity, there is not a direct correspondence between this analysis and the comparison of *η* at |**B**| = 0 and 0.5 *B*_0_ seen in Fig. 4, however for the p-block the sign of the change in value of *η* between |**B**| = 0 and 0.5 *B*_0_ generally aligns with that of the derivative of *η* at zero field.

Several general observations can be made from this analysis: the first is the similarity of the behaviour of atoms in a given group, secondly is that atoms belonging to a group with the highest value of the hardness in a period at zero field have a tendency to lower their hardness or at least keep it unchanged, whereas all other main group elements increase in hardness in a magnetic field. The same pattern of behaviour and similarity within a group is observed for the electronegativity. In a broader context, at least for the p-block elements, the trend of increasing *χ* from bottom left to top right becomes attenuated by a decrease in *χ* from left to right on application of an external magnetic field, whereas for the s-block elements the situation is less clear-cut.

From the present study on atoms, some insight may already be gained into the changes in chemistry that molecules may undergo in the presence of magnetic fields. The compression of the electronegativity and hardness scales that is observed at |**B**| = 0.5 *B*_0_ and the signs of their derivatives with respect to field strength at |**B**| = 0 indicate that the polarity of the bonds may be significantly affected by the magnetic field, to the point of reversing altogether, as may be deduced from the Huheey equation for charge transfer between two species.^74^ The effects predicted in this way may be compared with the effect of the magnetic field on the electron density and dipole moment of diatomic molecules.

## 5 Conclusions

The extension of the energy functional *E* = *E*(*N*, *v*) in conceptual DFT by inclusion of an external magnetic field has been investigated by studying the two most important global properties in conceptual DFT – the electronic chemical potential (electronegativity) and the hardness of atoms belonging to the main group elements up to Kr. Compared to previous work on the inclusion of electric fields and given the range of magnetic field strength considered (0.0–1.0 *B*_0_) the evolution of these quantities, and the quantities on which they depend in their Mulliken and Pearson definitions respectively, the ionisation energy and electron affinity, transpires to be much more complex. The reason for this is that the ground-state configuration of the atom, its anion and cation, changes as the magnetic field strength changes leading to discontinuities in their energies as functions of field strength. Since the changes in ground-state configuration of the three species for each element can occur at different field strengths, it can create a complicated piecewise structure for the electronegativity or hardness as a function of field strength. This is demonstrated for carbon at the Hartree–Fock level.

To compare with trends across the periodic table known at zero field for the hardness and electronegativity, a periodic table of *χ* and *η* values for the main group elements evaluated using current density functional theory at |**B**| = 0.5 *B*_0_ is presented. Both for the electronegativity and hardness, an overall increase in values is observed on the left side of the periodic table, whereas a decrease is seen on the right side, with a similar behavior of elements within the same column. The overall picture is a compression of the electronegativity and hardness range across each period, which would lead to important changes to be expected for bond polarity following Huheey's electronegativity equalization based approach.^74^

The derivatives of the electronegativity and hardness with respect to magnetic field strength at zero field, response functions in the conventional sense for conceptual DFT, present a simpler picture of the behaviour in a magnetic field since the changes in ground-state configuration do not need to be considered given the definition of the properties at zero field, thus more easily allowing a comparison across the periodic table. The behaviour of atoms within a group is seen to be similar both for *χ* and *η*, having derivatives with the same sign and order of magnitude for the electronegativity. For the p-block, the general picture is that the increase in *χ* from the bottom left to top right of the periodic table known at zero field becomes attenuated by the tendency of *χ* to decrease by an increasing magnitude from left to right across the periodic table in a magnetic field. For the hardness, the atoms in the group with the highest *η* in a period at zero field have a decreasing or unchanged hardness in the presence of a magnetic field, whilst all other elements have an increasing hardness in a magnetic field.

The present work focuses on chemical properties in static magnetic fields however further studies of the polarization of the density under non-uniform and oscillating magnetic fields (see, for example, ref. 75) may yield additional insight into this behavior.

## Data availability

The datasets supporting this article have been uploaded as part of the ESI.†

## Author contributions

R. F., T. J. P. I. and A. M. T. carried out all the calculations. A. M. T., F. D. P. and P. G. conceptualized the study, the study was supervised by A. M. T., T. J. P. I., F. D. P. and P. G. P. G. wrote the first draft of the paper, all authors contributed to analyzing the results and the writing, reviewing and editing towards the final version of the paper.

## Conflicts of interest

There are no conflicts to declare.

## Supplementary Material

SC-013-D1SC07263C-s001
