# Supplementary material for: Extending conceptual DFT to include external variables: the influence of magnetic fields
Source: Chem Sci. 2022 Apr 4;13(18):5311–24. doi: 10.1039/d1sc07263c (PMC9093152; doi:10.1039/d1sc07263c)
Supplement: SC-013-D1SC07263C-s001 [file SC-013-D1SC07263C-s001.pdf]

Electronic Supplementary Material (ESI) for Chemical Science.
This journal is © The Royal Society of Chemistry 2022

Extending Conceptual DFT to Include External Variables: The Influence of Magnetic Fields

Robin Francotte,[1] Tom J. P. Irons,[2] Andrew M. Teale,[2,3] Frank de Proft,[1,*] and Paul Geerlings[1]

[1]*Research Group of General Chemistry (ALGC), Vrije Universiteit Brussel (VUB), Pleinlaan 2, B-1050 Brussels, Belgium*
[2]*School of Chemistry, University of Nottingham, University Park, Nottingham, NG7 2RD, UK*
[3]*Hylleraas Centre for Quantum Molecular Sciences, Department of Chemistry, University of Oslo, P.O. Box 1033 Blindern, N-0315 Oslo, Norway*

[*] fdprof@vub.be

$\mathbf{B}$ / B_0	M_s	E_0 / E_h	M_s^-	E_0^- / E_h	M_s^+	E_0^+ / E_h	E.A. / E_h	I.P. / E_h	χ / E_h	η / E_h
0.0000	$-\frac{1}{2}$	-0.500229	0	-0.530768	0	-0.000000	0.030539	0.500229	0.265384	0.234845
0.0001	$-\frac{1}{2}$	-0.500279	0	-0.530768	0	-0.000000	0.030489	0.500279	0.265384	0.234895
0.0010	$-\frac{1}{2}$	-0.500729	0	-0.530766	0	-0.000000	0.030037	0.500729	0.265383	0.235346
0.0100	$-\frac{1}{2}$	-0.505203	0	-0.530508	0	-0.000000	0.025306	0.505203	0.265254	0.239949
0.0200	$-\frac{1}{2}$	-0.510125	0	-0.529775	0	-0.000000	0.019650	0.510125	0.264887	0.245237
0.0400	$-\frac{1}{2}$	-0.519813	0	-0.527303	0	-0.000000	0.007490	0.519813	0.263651	0.256162
0.0600	$-\frac{1}{2}$	-0.529296	-1	-0.524629	0	-0.000000	-0.004667	0.529296	0.262314	0.266981
0.0800	$-\frac{1}{2}$	-0.538576	-1	-0.537169	0	-0.000000	-0.001407	0.538576	0.268585	0.269991
0.1000	$-\frac{1}{2}$	-0.547658	-1	-0.546028	0	-0.000000	-0.001631	0.547658	0.273014	0.274645
0.2000	$-\frac{1}{2}$	-0.590292	-1	-0.587260	0	-0.000000	-0.003032	0.590292	0.293630	0.296662
0.3000	$-\frac{1}{2}$	-0.628852	-1	-0.638819	0	-0.000000	0.009966	0.628852	0.319409	0.309443
0.4000	$-\frac{1}{2}$	-0.664069	-1	-0.680867	0	-0.000000	0.016798	0.664069	0.340434	0.323636
0.5000	$-\frac{1}{2}$	-0.696539	-1	-0.714087	0	-0.000000	0.017548	0.696539	0.357043	0.339495
0.6000	$-\frac{1}{2}$	-0.726709	-1	-0.743598	0	-0.000000	0.016889	0.726709	0.371799	0.354910
0.7000	$-\frac{1}{2}$	-0.754919	-1	-0.773272	0	-0.000000	0.018353	0.754919	0.386636	0.368283
0.8000	$-\frac{1}{2}$	-0.781436	-1	-0.803412	0	-0.000000	0.021976	0.781436	0.401706	0.379730
0.9000	$-\frac{1}{2}$	-0.806482	-1	-0.832955	0	-0.000000	0.026473	0.806482	0.416478	0.390005
1.0000	$-\frac{1}{2}$	-0.830244	-1	-0.860928	0	-0.000000	0.030684	0.830244	0.430464	0.399780

TABLE I. Hydrogen Data

$\mathbf{B}$ / B_0	M_s	E_0 / E_h	M_s^-	E_0^- / E_h	M_s^+	E_0^+ / E_h	E.A. / E_h	I.P. / E_h	χ / E_h	η / E_h
0.0000	0	-2.909609	$-\frac{1}{2}$	-2.893192	$-\frac{1}{2}$	-2.000168	-0.016417	0.909441	0.446512	0.462929
0.0001	0	-2.909609	$-\frac{1}{2}$	-2.893242	$-\frac{1}{2}$	-2.000218	-0.016367	0.909391	0.446512	0.462879
0.0010	0	-2.909609	$-\frac{1}{2}$	-2.893685	$-\frac{1}{2}$	-2.000668	-0.015923	0.908941	0.446509	0.462432
0.0100	0	-2.909588	$-\frac{1}{2}$	-2.897516	$-\frac{1}{2}$	-2.005161	-0.012072	0.904427	0.446178	0.458250
0.0200	0	-2.909528	$-\frac{1}{2}$	-2.900492	$-\frac{1}{2}$	-2.010142	-0.009035	0.899385	0.445175	0.454210
0.0400	0	-2.909284	$-\frac{1}{2}$	-2.902427	$-\frac{1}{2}$	-2.020066	-0.006858	0.889219	0.441180	0.448038
0.0600	0	-2.908879	$-\frac{1}{2}$	-2.899108	$-\frac{1}{2}$	-2.029938	-0.009771	0.878941	0.434585	0.444356
0.0800	0	-2.908313	$-\frac{1}{2}$	-2.892339	$-\frac{1}{2}$	-2.039760	-0.015973	0.868552	0.426290	0.442263
0.1000	0	-2.907585	$-\frac{1}{2}$	-2.889753	$-\frac{1}{2}$	-2.049531	-0.017832	0.858054	0.420111	0.437943
0.2000	0	-2.901573	$-\frac{1}{2}$	-2.891162	$-\frac{1}{2}$	-2.097628	-0.010411	0.803946	0.396767	0.407178
0.3000	0	-2.891729	$-\frac{1}{2}$	-2.867742	$-\frac{1}{2}$	-2.144475	-0.023987	0.747255	0.361634	0.385621
0.4000	0	-2.878267	$-\frac{1}{2}$	-2.857872	$-\frac{1}{2}$	-2.190098	-0.020396	0.688169	0.333887	0.354283
0.5000	0	-2.861428	$-\frac{1}{2}$	-2.852356	$-\frac{1}{2}$	-2.234533	-0.009072	0.626895	0.308912	0.317984
0.6000	0	-2.841457	$-\frac{1}{2}$	-2.842299	$-\frac{1}{2}$	-2.277817	0.000841	0.563640	0.282241	0.281399
0.7000	0	-2.818596	$-\frac{1}{2}$	-2.825498	$-\frac{1}{2}$	-2.319996	0.006903	0.498600	0.252751	0.245848
0.8000	-1	-2.827407	$-\frac{3}{2}$	-2.854317	$-\frac{1}{2}$	-2.361113	0.026909	0.466294	0.246602	0.219693
0.9000	-1	-2.890396	$-\frac{3}{2}$	-2.927003	$-\frac{1}{2}$	-2.401213	0.036607	0.489182	0.262895	0.226288
1.0000	-1	-2.951930	$-\frac{3}{2}$	-2.995196	$-\frac{1}{2}$	-2.440342	0.043267	0.511588	0.277427	0.234160

TABLE II. Helium Data

$\mathbf{B}$ / B_0	M_s	E_0 / E_h	M_s^-	E_0^- / E_h	M_s^+	E_0^+ / E_h	E.A. / E_h	I.P. / E_h	χ / E_h	η / E_h
0.0000	$-\frac{1}{2}$	-7.488947	0	-7.510357	0	-7.286585	0.021410	0.202361	0.111886	0.090476
0.0001	$-\frac{1}{2}$	-7.488997	0	-7.510357	0	-7.286585	0.021360	0.202411	0.111886	0.090526
0.0010	$-\frac{1}{2}$	-7.489445	0	-7.510349	0	-7.286585	0.020904	0.202860	0.111882	0.090978
0.0100	$-\frac{1}{2}$	-7.493798	0	-7.509625	0	-7.286578	0.015827	0.207220	0.111524	0.095697
0.0200	$-\frac{1}{2}$	-7.498355	-1	-7.508996	0	-7.286555	0.010641	0.211800	0.111220	0.100580
0.0400	$-\frac{1}{2}$	-7.506613	-1	-7.527324	0	-7.286465	0.020711	0.220148	0.120429	0.099719
0.0600	$-\frac{1}{2}$	-7.513799	-1	-7.543655	0	-7.286314	0.029856	0.227485	0.128671	0.098814
0.0800	$-\frac{1}{2}$	-7.520016	-1	-7.557891	0	-7.286103	0.037875	0.233913	0.135894	0.098019
0.1000	$-\frac{1}{2}$	-7.525371	-1	-7.572005	0	-7.285833	0.046634	0.239539	0.143086	0.096452
0.2000	$-\frac{1}{2}$	-7.551610	-1	-7.616158	0	-7.283577	0.064548	0.268033	0.166290	0.101742
0.3000	$-\frac{1}{2}$	-7.590536	-1	-7.646027	0	-7.279826	0.055491	0.310710	0.183100	0.127610
0.4000	$-\frac{1}{2}$	-7.621849	-1	-7.667795	0	-7.274594	0.045946	0.347255	0.196600	0.150655
0.5000	$-\frac{1}{2}$	-7.647907	-1	-7.698177	0	-7.267896	0.050270	0.380011	0.215140	0.164871
0.6000	$-\frac{1}{2}$	-7.670119	-1	-7.723450	0	-7.259754	0.053330	0.410365	0.231848	0.178517
0.7000	$-\frac{1}{2}$	-7.689128	-1	-7.744362	0	-7.250193	0.055234	0.438935	0.247084	0.191851
0.8000	$-\frac{1}{2}$	-7.705152	-1	-7.761685	0	-7.239240	0.056533	0.465913	0.261223	0.204690
0.9000	$-\frac{1}{2}$	-7.718272	-1	-7.776032	0	-7.226922	0.057761	0.491350	0.274555	0.216795
1.0000	$-\frac{1}{2}$	-7.728601	-1	-7.787734	0	-7.213271	0.059133	0.515329	0.287231	0.228098

TABLE III. Lithium Data

$\mathbf{B}$ / B_0	M_s	E_0 / E_h	M_s^-	E_0^- / E_h	M_s^+	E_0^+ / E_h	E.A. / E_h	I.P. / E_h	χ / E_h	η / E_h
0.0000	0	-14.671421	$-\frac{1}{2}$	-14.667830	$-\frac{1}{2}$	-14.338435	-0.003591	0.332985	0.164697	0.168288
0.0001	0	-14.671421	$-\frac{1}{2}$	-14.667930	$-\frac{1}{2}$	-14.338485	-0.003491	0.332935	0.164722	0.168213
0.0010	0	-14.671419	$-\frac{1}{2}$	-14.668814	$-\frac{1}{2}$	-14.338935	-0.002605	0.332485	0.164940	0.167545
0.0100	0	-14.671282	$-\frac{1}{2}$	-14.676188	$-\frac{1}{2}$	-14.343382	0.004906	0.327900	0.166403	0.161497
0.0200	0	-14.670866	$-\frac{1}{2}$	-14.684611	$-\frac{1}{2}$	-14.348222	0.013744	0.322645	0.168194	0.154450
0.0400	0	-14.669211	$-\frac{1}{2}$	-14.693472	$-\frac{1}{2}$	-14.357581	0.024261	0.311629	0.167945	0.143684
0.0600	0	-14.666475	$-\frac{1}{2}$	-14.702628	$-\frac{1}{2}$	-14.366518	0.036153	0.299957	0.168055	0.131902
0.0800	-1	-14.687661	$-\frac{3}{2}$	-14.725720	$-\frac{1}{2}$	-14.375037	0.038059	0.312624	0.175341	0.137282
0.1000	-1	-14.711813	$-\frac{3}{2}$	-14.753846	$-\frac{1}{2}$	-14.383146	0.042033	0.328667	0.185350	0.143317
0.2000	-1	-14.817315	$-\frac{3}{2}$	-14.873141	$-\frac{1}{2}$	-14.417882	0.055825	0.399433	0.227629	0.171804
0.3000	-1	-14.902758	$-\frac{3}{2}$	-14.967020	$-\frac{1}{2}$	-14.438702	0.064262	0.464056	0.264159	0.199897
0.4000	-1	-14.973495	$-\frac{3}{2}$	-15.043943	$-\frac{1}{2}$	-14.500795	0.070448	0.472699	0.271574	0.201126
0.5000	-1	-15.033190	$-\frac{3}{2}$	-15.108258	$-\frac{1}{2}$	-14.556547	0.075068	0.476643	0.275856	0.200787
0.6000	-1	-15.084327	$-\frac{3}{2}$	-15.160861	$-\frac{1}{2}$	-14.607097	0.076534	0.477229	0.276882	0.200348
0.7000	-1	-15.128623	$-\frac{3}{2}$	-15.214865	$-\frac{1}{2}$	-14.653286	0.086242	0.475337	0.280789	0.194547
0.8000	-1	-15.167322	$-\frac{3}{2}$	-15.262172	$-\frac{1}{2}$	-14.695753	0.094851	0.471568	0.283209	0.188359
0.9000	-1	-15.201357	$-\frac{3}{2}$	-15.305858	$-\frac{1}{2}$	-14.734983	0.104500	0.466374	0.285437	0.180937
1.0000	-1	-15.223505	$-\frac{3}{2}$	-15.346982	$-\frac{1}{2}$	-14.771338	0.123477	0.452168	0.287822	0.164346

TABLE IV. Beryllium Data

$\mathbf{B}$ / B_0	M_s	E_0 / E_h	M_s^-	E_0^- / E_h	M_s^+	E_0^+ / E_h	E.A. / E_h	I.P. / E_h	χ / E_h	η / E_h
0.0000	$-\frac{1}{2}$	-24.669111	-1	-24.690025	0	-24.346824	0.020913	0.322288	0.171601	0.150687
0.0001	$-\frac{1}{2}$	-24.669161	-1	-24.690125	0	-24.346824	0.020963	0.322338	0.171650	0.150687
0.0010	$-\frac{1}{2}$	-24.669610	-1	-24.691032	0	-24.346823	0.021422	0.322787	0.172105	0.150683
0.0100	$-\frac{1}{2}$	-24.674001	-1	-24.701242	0	-24.346759	0.027241	0.327242	0.177242	0.150001
0.0200	$-\frac{1}{2}$	-24.678671	-1	-24.712776	0	-24.346565	0.034105	0.332106	0.183106	0.149000
0.0400	$-\frac{1}{2}$	-24.699454	-1	-24.736744	0	-24.345789	0.037290	0.353665	0.195478	0.158188
0.0600	$-\frac{1}{2}$	-24.716635	-1	-24.759938	0	-24.344497	0.043302	0.372139	0.207720	0.164418
0.0800	$-\frac{1}{2}$	-24.732724	-1	-24.781672	0	-24.342693	0.048948	0.390031	0.219490	0.170542
0.1000	$-\frac{1}{2}$	-24.747749	-1	-24.801911	0	-24.340381	0.054162	0.407369	0.230765	0.176604
0.2000	$-\frac{3}{2}$	-24.887169	-1	-24.898198	-1	-24.462897	0.011030	0.424271	0.217650	0.206621
0.3000	$-\frac{3}{2}$	-25.033054	-2	-25.056532	-1	-24.579159	0.023478	0.453895	0.238687	0.215208
0.4000	$-\frac{3}{2}$	-25.163112	-2	-25.208925	-1	-24.684211	0.045813	0.478901	0.262357	0.216544
0.5000	$-\frac{3}{2}$	-25.279954	-2	-25.344745	-1	-24.779456	0.064791	0.500498	0.282645	0.217853
0.6000	$-\frac{3}{2}$	-25.385680	-2	-25.464770	-1	-24.866141	0.079090	0.519539	0.299315	0.220225
0.7000	$-\frac{3}{2}$	-25.481958	-2	-25.574196	-1	-24.945330	0.092237	0.536628	0.314433	0.222196
0.8000	$-\frac{3}{2}$	-25.570103	-2	-25.676664	-1	-25.017923	0.106561	0.552180	0.329370	0.222809
0.9000	$-\frac{3}{2}$	-25.651161	-2	-25.773381	-1	-25.084687	0.122220	0.566474	0.344347	0.222127
1.0000	$-\frac{3}{2}$	-25.725990	-2	-25.864261	-1	-25.146284	0.138271	0.579706	0.358988	0.220718

TABLE V. Boron Data

$\mathbf{B}$ / B_0	M_s	E_0 / E_h	M_s^-	E_0^- / E_h	M_s^+	E_0^+ / E_h	E.A. / E_h	I.P. / E_h	χ / E_h	η / E_h
0.0000	-1	-37.860049	$-\frac{3}{2}$	-37.921269	$-\frac{1}{2}$	-37.445626	0.061220	0.414423	0.237821	0.176601
0.0001	-1	-37.860199	$-\frac{3}{2}$	-37.921419	$-\frac{1}{2}$	-37.445676	0.061220	0.414523	0.237871	0.176651
0.0010	-1	-37.861548	$-\frac{3}{2}$	-37.922766	$-\frac{1}{2}$	-37.446126	0.061218	0.415422	0.238320	0.177102
0.0100	-1	-37.874938	$-\frac{3}{2}$	-37.935949	$-\frac{1}{2}$	-37.450569	0.061011	0.424369	0.242690	0.181679
0.0200	-1	-37.889605	$-\frac{3}{2}$	-37.950017	$-\frac{1}{2}$	-37.455395	0.060412	0.434210	0.247311	0.186899
0.0400	-1	-37.918275	$-\frac{3}{2}$	-37.976582	$-\frac{1}{2}$	-37.464700	0.058307	0.453575	0.255941	0.197634
0.0600	-1	-37.946064	$-\frac{3}{2}$	-38.001398	$-\frac{1}{2}$	-37.492393	0.055334	0.453671	0.254502	0.199169
0.0800	-1	-37.972982	$-\frac{3}{2}$	-38.024637	$-\frac{1}{2}$	-37.510402	0.051655	0.462580	0.257118	0.205463
0.1000	-1	-37.999040	$-\frac{3}{2}$	-38.046373	$-\frac{1}{2}$	-37.527847	0.047333	0.471193	0.259263	0.211930
0.2000	-1	-38.116950	$-\frac{1}{2}$	-38.075610	$-\frac{3}{2}$	-37.623498	-0.041340	0.493453	0.226056	0.267396
0.3000	-1	-38.215775	$-\frac{3}{2}$	-38.224657	$-\frac{3}{2}$	-37.793600	0.008882	0.422175	0.215529	0.206647
0.4000	-2	-38.334059	$-\frac{5}{2}$	-38.354030	$-\frac{3}{2}$	-37.952937	0.019971	0.381122	0.200546	0.180575
0.5000	-2	-38.459363	$-\frac{5}{2}$	-38.496627	$-\frac{3}{2}$	-38.102363	0.037263	0.357001	0.197132	0.159869
0.6000	-2	-38.625277	$-\frac{5}{2}$	-38.662772	$-\frac{3}{2}$	-38.242717	0.037495	0.382560	0.210027	0.172533
0.7000	-2	-38.794228	$-\frac{5}{2}$	-38.831527	$-\frac{3}{2}$	-38.374789	0.037298	0.419440	0.228369	0.191071
0.8000	-2	-38.951647	$-\frac{5}{2}$	-38.990267	$-\frac{3}{2}$	-38.499302	0.038620	0.452345	0.245483	0.206863
0.9000	-2	-39.099500	$-\frac{5}{2}$	-39.144492	$-\frac{3}{2}$	-38.616908	0.044993	0.482592	0.263792	0.218799
1.0000	-2	-39.239738	$-\frac{5}{2}$	-39.295516	$-\frac{3}{2}$	-38.728188	0.055778	0.511550	0.283664	0.227886

TABLE VI. Carbon Data

$\mathbf{B}$ / B_0	M_s	E_0 / E_h	M_s^-	E_0^- / E_h	M_s^+	E_0^+ / E_h	E.A. / E_h	I.P. / E_h	χ / E_h	η / E_h
0.0000	$-\frac{3}{2}$	-54.615841	-1	-54.629686	-1	-54.068139	0.013845	0.547702	0.280773	0.266928
0.0001	$-\frac{3}{2}$	-54.615991	-1	-54.629786	-1	-54.068289	0.013795	0.547702	0.280748	0.266953
0.0010	$-\frac{3}{2}$	-54.617340	-1	-54.630683	-1	-54.069639	0.013343	0.547701	0.280522	0.267179
0.0100	$-\frac{3}{2}$	-54.630737	-1	-54.638526	-1	-54.083078	0.007789	0.547660	0.277724	0.269936
0.0200	$-\frac{3}{2}$	-54.645426	-1	-54.651516	-1	-54.097892	0.006090	0.547535	0.276812	0.270722
0.0400	$-\frac{3}{2}$	-54.674183	-1	-54.674478	-1	-54.127148	0.000296	0.547034	0.273665	0.273369
0.0600	$-\frac{3}{2}$	-54.702114	-2	-54.699427	-1	-54.155910	-0.002686	0.546203	0.271758	0.274445
0.0800	$-\frac{3}{2}$	-54.729224	-2	-54.718759	-1	-54.184179	-0.010465	0.545045	0.267290	0.277755
0.1000	$-\frac{3}{2}$	-54.755520	-2	-54.744202	-1	-54.211955	-0.011319	0.543565	0.266123	0.277442
0.2000	$-\frac{3}{2}$	-54.875104	-2	-54.863041	-1	-54.343531	-0.012063	0.531573	0.259755	0.271818
0.3000	$-\frac{3}{2}$	-54.975874	-2	-54.969332	-1	-54.463225	-0.006542	0.512649	0.253054	0.259596
0.4000	$-\frac{3}{2}$	-55.059341	-2	-55.065105	-1	-54.571551	0.005764	0.487790	0.246777	0.241013
0.5000	$-\frac{3}{2}$	-55.127004	-2	-55.139421	-2	-54.699450	0.012417	0.427554	0.219985	0.207568
0.6000	$-\frac{5}{2}$	-55.193878	-3	-55.210637	-2	-54.834805	0.016759	0.359073	0.187916	0.171157
0.7000	$-\frac{5}{2}$	-55.358332	-3	-55.384038	-2	-54.960446	0.025707	0.397886	0.211796	0.186089
0.8000	$-\frac{5}{2}$	-55.513116	-3	-55.544132	-2	-55.077019	0.031016	0.436097	0.233557	0.202540
0.9000	$-\frac{5}{2}$	-55.657180	-3	-55.689185	-2	-55.188382	0.032005	0.468799	0.250402	0.218397
1.0000	$-\frac{5}{2}$	-55.790350	-3	-55.821914	-2	-55.328589	0.031564	0.461761	0.246662	0.215098

TABLE VII. Nitrogen Data

$\mathbf{B}$ / B_0	M_s	E_0 / E_h	M_s^-	E_0^- / E_h	M_s^+	E_0^+ / E_h	E.A. / E_h	I.P. / E_h	χ / E_h	η / E_h
0.0000	-1	-75.095994	$-\frac{1}{2}$	-75.157431	$-\frac{3}{2}$	-74.593425	0.061438	0.502568	0.282003	0.220565
0.0001	-1	-75.096144	$-\frac{1}{2}$	-75.157531	$-\frac{3}{2}$	-74.593575	0.061388	0.502568	0.281978	0.220590
0.0010	-1	-75.097493	$-\frac{1}{2}$	-75.158429	$-\frac{3}{2}$	-74.594925	0.060936	0.502568	0.281752	0.220816
0.0100	-1	-75.110893	$-\frac{1}{2}$	-75.167200	$-\frac{3}{2}$	-74.608363	0.056307	0.502529	0.279418	0.223111
0.0200	-1	-75.125591	$-\frac{1}{2}$	-75.176523	$-\frac{3}{2}$	-74.623178	0.050933	0.502413	0.276673	0.225740
0.0400	-1	-75.154383	$-\frac{1}{2}$	-75.193983	$-\frac{3}{2}$	-74.652434	0.039600	0.501949	0.270774	0.231174
0.0600	-1	-75.182373	$-\frac{1}{2}$	-75.210092	$-\frac{3}{2}$	-74.681196	0.027719	0.501177	0.264448	0.236729
0.0800	-1	-75.209565	$-\frac{1}{2}$	-75.225005	$-\frac{3}{2}$	-74.709464	0.015440	0.500101	0.257771	0.242330
0.1000	-1	-75.235964	$-\frac{1}{2}$	-75.238767	$-\frac{3}{2}$	-74.737238	0.002804	0.498725	0.250765	0.247961
0.2000	-1	-75.356316	$-\frac{1}{2}$	-75.332552	$-\frac{3}{2}$	-74.868765	-0.023764	0.487551	0.231894	0.255658
0.3000	-1	-75.458086	$-\frac{3}{2}$	-75.438611	$-\frac{3}{2}$	-74.988254	-0.019475	0.469832	0.225179	0.244653
0.4000	-1	-75.542498	$-\frac{3}{2}$	-75.529177	$-\frac{3}{2}$	-75.096075	-0.013321	0.446423	0.216551	0.229872
0.5000	-1	-75.610792	$-\frac{3}{2}$	-75.610082	$-\frac{3}{2}$	-75.192676	-0.000709	0.418115	0.208703	0.209412
0.6000	-1	-75.664139	$-\frac{3}{2}$	-75.672844	$-\frac{3}{2}$	-75.278548	0.008705	0.385591	0.197148	0.188443
0.7000	-2	-75.731232	$-\frac{5}{2}$	-75.740935	$-\frac{3}{2}$	-75.354196	0.009703	0.377036	0.193369	0.183666
0.8000	-2	-75.830378	$-\frac{5}{2}$	-75.848073	$-\frac{3}{2}$	-75.420125	0.017696	0.410252	0.213974	0.196278
0.9000	-2	-75.921119	$-\frac{5}{2}$	-75.944940	$-\frac{3}{2}$	-75.476828	0.023821	0.444291	0.234056	0.210235
1.0000	-2	-76.003071	$-\frac{5}{2}$	-76.029130	$-\frac{5}{2}$	-75.566263	0.026059	0.436808	0.231433	0.205374

TABLE VIII. Oxygen Data

**B** / B_0	M_s	E_0 / E_h	M_s^-	E_0^- / E_h	M_s^+	E_0^+ / E_h	E.A. / E_h	I.P. / E_h	χ / E_h	η / E_h
0.0000	$-\frac{1}{2}$	-99.767617	0	-99.905781	-1	-99.121835	0.138164	0.645782	0.391973	0.253809
0.0001	$-\frac{1}{2}$	-99.767717	0	-99.905781	-1	-99.121985	0.138064	0.645732	0.391898	0.253834
0.0010	$-\frac{1}{2}$	-99.768616	0	-99.905779	-1	-99.123334	0.137164	0.645282	0.391223	0.254059
0.0100	$-\frac{1}{2}$	-99.777531	0	-99.905621	-1	-99.136772	0.128091	0.640758	0.384425	0.256334
0.0200	$-\frac{1}{2}$	-99.787273	0	-99.905145	-1	-99.151586	0.117871	0.635687	0.376779	0.258908
0.0400	$-\frac{1}{2}$	-99.806244	0	-99.903253	-1	-99.180839	0.097009	0.625405	0.361207	0.264198
0.0600	$-\frac{1}{2}$	-99.824529	0	-99.900146	-1	-99.209595	0.075617	0.614934	0.345276	0.269659
0.0800	$-\frac{1}{2}$	-99.842132	0	-99.895872	-1	-99.237855	0.053740	0.604278	0.329009	0.275269
0.1000	$-\frac{1}{2}$	-99.859055	0	-99.890470	-1	-99.265618	0.031415	0.593437	0.312426	0.281011
0.2000	$-\frac{1}{2}$	-99.933602	-1	-99.902402	-1	-99.397038	-0.031201	0.536564	0.252682	0.283882
0.3000	$-\frac{1}{2}$	-99.991852	-1	-99.979629	-1	-99.516293	-0.012223	0.475559	0.231668	0.243891
0.4000	$-\frac{1}{2}$	-100.034569	-1	-100.011130	-1	-99.623683	-0.023439	0.410887	0.193724	0.217163
0.5000	$-\frac{1}{2}$	-100.062574	-1	-100.045058	-1	-99.719575	-0.017516	0.342999	0.162742	0.180258
0.6000	$-\frac{3}{2}$	-100.133534	-2	-100.139057	-1	-99.804377	0.005523	0.329156	0.167340	0.161817
0.7000	$-\frac{3}{2}$	-100.237962	-2	-100.246118	-1	-99.878518	0.008156	0.359444	0.183800	0.175644
0.8000	$-\frac{3}{2}$	-100.329712	-2	-100.326545	-1	-99.942426	-0.003167	0.387287	0.192060	0.195227
0.9000	$-\frac{3}{2}$	-100.411256	-2	-100.404655	-1	-99.996527	-0.006601	0.414729	0.204064	0.210665
1.0000	$-\frac{3}{2}$	-100.484175	-2	-100.481319	-1	-100.041236	-0.002855	0.442939	0.220042	0.222897

TABLE IX. Fluorine Data

**B** / B_0	M_s	E_0 / E_h	M_s^-	E_0^- / E_h	M_s^+	E_0^+ / E_h	E.A. / E_h	I.P. / E_h	χ / E_h	η / E_h
0.0000	0	-128.980517	$-\frac{1}{2}$	-128.939603	$-\frac{1}{2}$	-128.177003	-0.040913	0.803514	0.381300	0.422214
0.0001	0	-128.980517	$-\frac{1}{2}$	-128.939653	$-\frac{1}{2}$	-128.177103	-0.040863	0.803414	0.381275	0.422139
0.0010	0	-128.980516	$-\frac{1}{2}$	-128.940099	$-\frac{1}{2}$	-128.178002	-0.040416	0.802514	0.381049	0.421465
0.0100	0	-128.980435	$-\frac{1}{2}$	-128.944224	$-\frac{1}{2}$	-128.186947	-0.036211	0.793488	0.378638	0.414850
0.0200	0	-128.980191	$-\frac{1}{2}$	-128.956322	$-\frac{1}{2}$	-128.196780	-0.023869	0.783412	0.379771	0.403640
0.0400	0	-128.979217	$-\frac{1}{2}$	-128.967133	$-\frac{1}{2}$	-128.216111	-0.012084	0.763106	0.375511	0.387595
0.0600	0	-128.977593	$-\frac{1}{2}$	-128.972176	$-\frac{1}{2}$	-128.234996	-0.005417	0.742597	0.368590	0.374007
0.0800	0	-128.975321	$-\frac{1}{2}$	-128.971645	$-\frac{1}{2}$	-128.253436	-0.003676	0.721885	0.359105	0.362780
0.1000	0	-128.972404	$-\frac{1}{2}$	-128.966418	$-\frac{1}{2}$	-128.271431	-0.005986	0.700972	0.347493	0.353479
0.2000	0	-128.948224	$-\frac{1}{2}$	-128.914741	$-\frac{1}{2}$	-128.354760	-0.033482	0.593464	0.279991	0.313473
0.3000	0	-128.908397	$-\frac{1}{2}$	-128.861140	$-\frac{1}{2}$	-128.427113	-0.047257	0.481284	0.217013	0.264270
0.4000	0	-128.853493	$-\frac{1}{2}$	-128.828245	$-\frac{1}{2}$	-128.488684	-0.025249	0.364810	0.169780	0.195029
0.5000	-1	-128.824638	$-\frac{3}{2}$	-128.846937	$-\frac{1}{2}$	-128.539716	0.022299	0.284922	0.153610	0.131311
0.6000	-1	-128.899626	$-\frac{3}{2}$	-128.909295	$-\frac{1}{2}$	-128.580491	0.009669	0.319135	0.164402	0.154733
0.7000	-1	-128.964026	$-\frac{3}{2}$	-128.985979	$-\frac{1}{2}$	-128.611309	0.021952	0.352718	0.187335	0.165383
0.8000	-1	-129.015672	$-\frac{3}{2}$	-129.028577	$-\frac{1}{2}$	-128.632484	0.012905	0.383189	0.198047	0.185142
0.9000	-1	-129.054897	$-\frac{3}{2}$	-129.053069	$-\frac{1}{2}$	-128.644331	-0.001828	0.410566	0.204369	0.206197
1.0000	-1	-129.083255	$-\frac{3}{2}$	-129.068948	$-\frac{1}{2}$	-128.647165	-0.014308	0.436090	0.210891	0.225199

TABLE X. Neon Data

$\mathbf{B}$ / B_0	M_s	E_0 / E_h	M_s^-	E_0^- / E_h	M_s^+	E_0^+ / E_h	E.A. / E_h	I.P. / E_h	χ / E_h	η / E_h
0.0000	$-\frac{1}{2}$	-162.297921	0	-162.319420	0	-162.107544	0.021499	0.190377	0.105938	0.084439
0.0001	$-\frac{1}{2}$	-162.297971	0	-162.319420	0	-162.107544	0.021449	0.190427	0.105938	0.084489
0.0010	$-\frac{1}{2}$	-162.298419	0	-162.319411	0	-162.107543	0.020992	0.190876	0.105934	0.084942
0.0100	$-\frac{1}{2}$	-162.302703	0	-162.318583	0	-162.107489	0.015880	0.195214	0.105547	0.089667
0.0200	$-\frac{1}{2}$	-162.307051	0	-162.316398	0	-162.107325	0.009347	0.199726	0.104537	0.095189
0.0400	$-\frac{1}{2}$	-162.314485	-1	-162.331533	0	-162.106670	0.017047	0.207815	0.112431	0.095384
0.0600	$-\frac{1}{2}$	-162.320334	-1	-162.344853	0	-162.105578	0.024519	0.214755	0.119637	0.095118
0.0800	$-\frac{1}{2}$	-162.324733	-1	-162.355279	0	-162.104050	0.030546	0.220683	0.125615	0.095069
0.1000	$-\frac{1}{2}$	-162.327821	-1	-162.363302	0	-162.102085	0.035481	0.225736	0.130608	0.095128
0.2000	$-\frac{1}{2}$	-162.327415	-1	-162.378853	0	-162.085742	0.051438	0.241673	0.146556	0.095118
0.3000	$-\frac{1}{2}$	-162.307032	-1	-162.366089	0	-162.058606	0.059056	0.248427	0.153741	0.094685
0.4000	$-\frac{1}{2}$	-162.271453	-1	-162.357571	0	-162.020824	0.086118	0.250630	0.168374	0.082256
0.5000	$-\frac{1}{2}$	-162.262679	-1	-162.329527	0	-161.972581	0.066848	0.290098	0.178473	0.111625
0.6000	$-\frac{1}{2}$	-162.230677	-1	-162.288527	0	-161.914097	0.057849	0.316580	0.187215	0.129365
0.7000	$-\frac{1}{2}$	-162.187371	-1	-162.234819	0	-161.845609	0.047448	0.341763	0.194605	0.147157
0.8000	$-\frac{1}{2}$	-162.132637	-1	-162.193861	0	-161.767366	0.061224	0.365272	0.213248	0.152024
0.9000	$-\frac{3}{2}$	-162.179079	-2	-162.293822	-1	-161.751055	0.114743	0.428024	0.271383	0.156641
1.0000	$-\frac{3}{2}$	-162.236609	-2	-162.359666	-1	-161.809480	0.123057	0.427129	0.275093	0.152036

TABLE XI. Sodium Data

$\mathbf{B}$ / B_0	M_s	E_0 / E_h	M_s^-	E_0^- / E_h	M_s^+	E_0^+ / E_h	E.A. / E_h	I.P. / E_h	χ / E_h	η / E_h
0.0000	0	-200.092463	$-\frac{1}{2}$	-200.085657	$-\frac{1}{2}$	-199.815123	-0.006806	0.277340	0.135267	0.142073
0.0001	0	-200.092463	$-\frac{1}{2}$	-200.085757	$-\frac{1}{2}$	-199.815173	-0.006706	0.277290	0.135292	0.141998
0.0010	0	-200.092461	$-\frac{1}{2}$	-200.086636	$-\frac{1}{2}$	-199.815621	-0.005825	0.276839	0.135507	0.141332
0.0100	0	-200.092226	$-\frac{1}{2}$	-200.093540	$-\frac{1}{2}$	-199.820008	0.001314	0.272218	0.136766	0.135452
0.0200	0	-200.091515	$-\frac{1}{2}$	-200.102117	$-\frac{1}{2}$	-199.824664	0.010602	0.266851	0.138727	0.128125
0.0400	0	-200.088691	$-\frac{1}{2}$	-200.109734	$-\frac{1}{2}$	-199.833291	0.021043	0.255399	0.138221	0.117178
0.0600	0	-200.084038	$-\frac{1}{2}$	-200.115727	$-\frac{1}{2}$	-199.841014	0.031689	0.243024	0.137357	0.105667
0.0800	-1	-200.095877	$-\frac{3}{2}$	-200.132636	$-\frac{1}{2}$	-199.847846	0.036759	0.248031	0.142395	0.105636
0.1000	-1	-200.115292	$-\frac{3}{2}$	-200.155009	$-\frac{1}{2}$	-199.853806	0.039718	0.261485	0.150602	0.110884
0.2000	-1	-200.186167	$-\frac{3}{2}$	-200.237071	$-\frac{1}{2}$	-199.871364	0.050904	0.314803	0.182853	0.131949
0.3000	-1	-200.222985	$-\frac{3}{2}$	-200.302060	$-\frac{1}{2}$	-199.870875	0.079075	0.352110	0.215593	0.136517
0.4000	-1	-200.234475	$-\frac{3}{2}$	-200.341638	$-\frac{1}{2}$	-199.855296	0.107163	0.379179	0.243171	0.136008
0.5000	-1	-200.226095	$-\frac{3}{2}$	-200.358248	$-\frac{1}{2}$	-199.826955	0.132154	0.399140	0.265647	0.133493
0.6000	-1	-200.241608	$-\frac{3}{2}$	-200.357594	$-\frac{1}{2}$	-199.811949	0.115986	0.429659	0.272822	0.156837
0.7000	-1	-200.245038	$-\frac{3}{2}$	-200.343314	$-\frac{1}{2}$	-199.808625	0.098276	0.436414	0.267345	0.169069
0.8000	-1	-200.235257	$-\frac{3}{2}$	-200.331777	$-\frac{1}{2}$	-199.796114	0.096520	0.439143	0.267831	0.171312
0.9000	-1	-200.212923	$-\frac{3}{2}$	-200.316280	$-\frac{1}{2}$	-199.773622	0.103357	0.439301	0.271329	0.167972
1.0000	-1	-200.178797	$-\frac{3}{2}$	-200.288641	$-\frac{1}{2}$	-199.741714	0.109844	0.437083	0.273463	0.163619

TABLE XII. Magnesium Data

$\mathbf{B}$ / B_0	M_s	E_0 / E_h	M_s^-	E_0^- / E_h	M_s^+	E_0^+ / E_h	E.A. / E_h	I.P. / E_h	χ / E_h	η / E_h
0.0000	$-\frac{1}{2}$	-242.383976	-1	-242.408395	0	-242.161800	0.024419	0.222176	0.123298	0.098879
0.0001	$-\frac{1}{2}$	-242.384076	-1	-242.408495	0	-242.161800	0.024419	0.222276	0.123348	0.098929
0.0010	$-\frac{1}{2}$	-242.384973	-1	-242.409380	0	-242.161799	0.024407	0.223175	0.123791	0.099384
0.0100	$-\frac{1}{2}$	-242.393684	-1	-242.419570	0	-242.161659	0.025886	0.232024	0.128955	0.103069
0.0200	$-\frac{1}{2}$	-242.402807	-1	-242.432077	0	-242.161237	0.029270	0.241570	0.135420	0.106150
0.0400	$-\frac{1}{2}$	-242.419321	-1	-242.455032	0	-242.159550	0.035711	0.259772	0.147741	0.112030
0.0600	$-\frac{1}{2}$	-242.433580	-1	-242.474889	0	-242.156746	0.041309	0.276835	0.159072	0.117763
0.0800	$-\frac{1}{2}$	-242.445666	-1	-242.491763	0	-242.152835	0.046096	0.292831	0.169464	0.123367
0.1000	$-\frac{1}{2}$	-242.455672	-1	-242.505864	0	-242.147835	0.050192	0.307838	0.179015	0.128823
0.2000	$-\frac{3}{2}$	-242.558382	-2	-242.619226	-1	-242.231157	0.060844	0.327225	0.194035	0.133191
0.3000	$-\frac{3}{2}$	-242.650002	-2	-242.753581	-1	-242.303651	0.103579	0.346351	0.224965	0.121386
0.4000	$-\frac{3}{2}$	-242.740887	-2	-242.853789	-1	-242.352492	0.112902	0.388395	0.250648	0.137747
0.5000	$-\frac{3}{2}$	-242.821191	-2	-242.928214	-1	-242.381355	0.107023	0.439836	0.273430	0.166406
0.6000	$-\frac{3}{2}$	-242.880949	-2	-242.982113	-1	-242.393109	0.101164	0.487840	0.294502	0.193338
0.7000	$-\frac{3}{2}$	-242.922563	-2	-243.017776	-1	-242.389884	0.095213	0.532679	0.313946	0.218733
0.8000	$-\frac{3}{2}$	-242.948009	-2	-243.058319	-1	-242.422464	0.110310	0.525545	0.317927	0.207618
0.9000	$-\frac{3}{2}$	-242.959265	-2	-243.085670	-1	-242.446477	0.126405	0.512788	0.319597	0.193191
1.0000	$-\frac{3}{2}$	-242.958211	-2	-243.100099	-1	-242.458669	0.141888	0.499542	0.320715	0.178827

TABLE XIII. Aluminium Data

$\mathbf{B}$ / B_0	M_s	E_0 / E_h	M_s^-	E_0^- / E_h	M_s^+	E_0^+ / E_h	E.A. / E_h	I.P. / E_h	χ / E_h	η / E_h
0.0000	-1	-289.396414	$-\frac{3}{2}$	-289.452742	$-\frac{1}{2}$	-289.093102	0.056328	0.303313	0.179820	0.123493
0.0001	-1	-289.396564	$-\frac{3}{2}$	-289.452892	$-\frac{1}{2}$	-289.093201	0.056328	0.303363	0.179845	0.123518
0.0010	-1	-289.397912	$-\frac{3}{2}$	-289.454237	$-\frac{1}{2}$	-289.094100	0.056325	0.303812	0.180069	0.123744
0.0100	-1	-289.411164	$-\frac{3}{2}$	-289.467235	$-\frac{1}{2}$	-289.102832	0.056071	0.308332	0.182201	0.126130
0.0200	-1	-289.425414	$-\frac{3}{2}$	-289.480746	$-\frac{1}{2}$	-289.112399	0.055332	0.313015	0.184174	0.128842
0.0400	-1	-289.452421	$-\frac{3}{2}$	-289.505059	$-\frac{1}{2}$	-289.130293	0.052638	0.322128	0.187383	0.134745
0.0600	-1	-289.477461	$-\frac{3}{2}$	-289.526027	$-\frac{1}{2}$	-289.146792	0.048566	0.330669	0.189618	0.141051
0.0800	-1	-289.500569	$-\frac{3}{2}$	-289.543874	$-\frac{1}{2}$	-289.161909	0.043305	0.338661	0.190983	0.147678
0.1000	-1	-289.521792	$-\frac{3}{2}$	-289.558800	$-\frac{1}{2}$	-289.175658	0.037009	0.346134	0.191571	0.154563
0.2000	-1	-289.601461	$-\frac{3}{2}$	-289.649610	$-\frac{3}{2}$	-289.243426	0.048149	0.358035	0.203092	0.154943
0.3000	-2	-289.733353	$-\frac{5}{2}$	-289.764097	$-\frac{3}{2}$	-289.368407	0.030744	0.364946	0.197845	0.167101
0.4000	-2	-289.901992	$-\frac{5}{2}$	-289.954747	$-\frac{3}{2}$	-289.468373	0.052755	0.433620	0.243187	0.190432
0.5000	-2	-290.041989	$-\frac{5}{2}$	-290.107041	$-\frac{3}{2}$	-289.559609	0.065053	0.482380	0.273716	0.208663
0.6000	-2	-290.158105	$-\frac{5}{2}$	-290.246330	$-\frac{3}{2}$	-289.672028	0.088225	0.486077	0.287151	0.198926
0.7000	-2	-290.254105	$-\frac{5}{2}$	-290.362305	$-\frac{3}{2}$	-289.765815	0.108200	0.488289	0.298245	0.190045
0.8000	-2	-290.332540	$-\frac{5}{2}$	-290.458834	$-\frac{3}{2}$	-289.843030	0.126294	0.489510	0.307902	0.181608
0.9000	-2	-290.395105	$-\frac{5}{2}$	-290.539520	$-\frac{3}{2}$	-289.905193	0.144414	0.489912	0.317163	0.172749
1.0000	-2	-290.443067	$-\frac{5}{2}$	-290.606720	$-\frac{3}{2}$	-289.953570	0.163654	0.489497	0.326575	0.162922

TABLE XIV. Silicon Data

**B** / B_0	M_s	E_0 / E_h	M_s^-	E_0^- / E_h	M_s^+	E_0^+ / E_h	E.A. / E_h	I.P. / E_h	χ / E_h	η / E_h
0.0000	$-\frac{3}{2}$	-341.295635	-1	-341.323105	-1	-340.905986	0.027470	0.389649	0.208559	0.181089
0.0001	$-\frac{3}{2}$	-341.295785	-1	-341.323255	-1	-340.906136	0.027470	0.389649	0.208559	0.181089
0.0010	$-\frac{3}{2}$	-341.297132	-1	-341.324599	-1	-340.907484	0.027467	0.389648	0.208557	0.181090
0.0100	$-\frac{3}{2}$	-341.310384	-1	-341.337556	-1	-340.920823	0.027172	0.389561	0.208366	0.181195
0.0200	$-\frac{3}{2}$	-341.324632	-1	-341.350982	-1	-340.935333	0.026350	0.389299	0.207824	0.181474
0.0400	$-\frac{3}{2}$	-341.351629	-1	-341.375241	-1	-340.963377	0.023612	0.388253	0.205932	0.182320
0.0600	$-\frac{3}{2}$	-341.376641	-1	-341.396409	-1	-340.990120	0.019769	0.386521	0.203145	0.183376
0.0800	$-\frac{3}{2}$	-341.399690	-1	-341.414571	-1	-341.015570	0.014881	0.384120	0.199501	0.184619
0.1000	$-\frac{3}{2}$	-341.420808	-1	-341.429842	-1	-341.039737	0.009035	0.381071	0.195053	0.186018
0.2000	$-\frac{3}{2}$	-341.498742	-2	-341.539084	-1	-341.141789	0.040343	0.356953	0.198648	0.158305
0.3000	$-\frac{3}{2}$	-341.577930	-2	-341.622734	-1	-341.214133	0.044804	0.363797	0.204300	0.159496
0.4000	$-\frac{5}{2}$	-341.697644	-3	-341.755087	-2	-341.356412	0.057443	0.341232	0.199338	0.141895
0.5000	$-\frac{5}{2}$	-341.921937	-3	-341.991731	-2	-341.548801	0.069794	0.373136	0.221465	0.151671
0.6000	$-\frac{5}{2}$	-342.122109	-3	-342.213835	-2	-341.717827	0.091727	0.404282	0.248004	0.156277
0.7000	$-\frac{5}{2}$	-342.298366	-3	-342.413612	-2	-341.866223	0.115246	0.432143	0.273695	0.158449
0.8000	$-\frac{5}{2}$	-342.453663	-3	-342.590988	-2	-341.996273	0.137325	0.457390	0.297358	0.160033
0.9000	$-\frac{5}{2}$	-342.590421	-3	-342.747436	-2	-342.109797	0.157015	0.480624	0.318820	0.161805
1.0000	$-\frac{5}{2}$	-342.710552	-3	-342.885684	-2	-342.208232	0.175132	0.502320	0.338726	0.163594

TABLE XV. Phosphorous Data

**B** / B_0	M_s	E_0 / E_h	M_s^-	E_0^- / E_h	M_s^+	E_0^+ / E_h	E.A. / E_h	I.P. / E_h	χ / E_h	η / E_h
0.0000	-1	-398.142240	$-\frac{1}{2}$	-398.220750	$-\frac{3}{2}$	-397.763747	0.078510	0.378493	0.228502	0.149992
0.0001	-1	-398.142390	$-\frac{1}{2}$	-398.220850	$-\frac{3}{2}$	-397.763897	0.078460	0.378493	0.228477	0.150017
0.0010	-1	-398.143738	$-\frac{1}{2}$	-398.221747	$-\frac{3}{2}$	-397.765245	0.078009	0.378493	0.228251	0.150242
0.0100	-1	-398.156989	$-\frac{1}{2}$	-398.230383	$-\frac{3}{2}$	-397.778575	0.073394	0.378414	0.225904	0.152510
0.0200	-1	-398.171236	$-\frac{1}{2}$	-398.239287	$-\frac{3}{2}$	-397.793061	0.068050	0.378175	0.223113	0.155062
0.0400	-1	-398.198228	$-\frac{1}{2}$	-398.254947	$-\frac{3}{2}$	-397.821006	0.056718	0.377222	0.216970	0.160252
0.0600	-1	-398.223228	$-\frac{1}{2}$	-398.267830	$-\frac{3}{2}$	-397.847585	0.044602	0.375643	0.210123	0.165520
0.0800	-1	-398.246253	$-\frac{1}{2}$	-398.278037	$-\frac{3}{2}$	-397.872802	0.031784	0.373451	0.202617	0.170833
0.1000	-1	-398.267327	$-\frac{1}{2}$	-398.285663	$-\frac{3}{2}$	-397.896665	0.018336	0.370662	0.194499	0.176163
0.2000	-1	-398.344493	$-\frac{3}{2}$	-398.368811	$-\frac{3}{2}$	-397.996031	0.024318	0.348462	0.186390	0.162072
0.3000	-2	-398.436767	$-\frac{5}{2}$	-398.461419	$-\frac{3}{2}$	-398.063427	0.024651	0.373340	0.198996	0.174344
0.4000	-2	-398.554085	$-\frac{5}{2}$	-398.597138	$-\frac{3}{2}$	-398.136694	0.043053	0.417392	0.230222	0.187170
0.5000	-2	-398.652763	$-\frac{5}{2}$	-398.711530	$-\frac{3}{2}$	-398.277799	0.058767	0.374964	0.216866	0.158099
0.6000	-3	-398.863778	$-\frac{7}{2}$	-398.931540	$-\frac{5}{2}$	-398.488980	0.067762	0.374798	0.221280	0.153518
0.7000	-3	-399.113768	$-\frac{7}{2}$	-399.154818	$-\frac{5}{2}$	-398.724377	0.041050	0.389391	0.215221	0.174170
0.8000	-3	-399.367730	$-\frac{7}{2}$	-399.418268	$-\frac{5}{2}$	-398.938880	0.050538	0.428850	0.239694	0.189156
0.9000	-3	-399.600810	$-\frac{7}{2}$	-399.657486	$-\frac{5}{2}$	-399.134461	0.056675	0.466350	0.261513	0.204837
1.0000	-3	-399.814261	$-\frac{7}{2}$	-399.872826	$-\frac{5}{2}$	-399.312803	0.058566	0.501457	0.280011	0.221446

TABLE XVI. Sulfur Data

**B** / B_0	M_s	E_0 / E_h	M_s^-	E_0^- / E_h	M_s^+	E_0^+ / E_h	E.A. / E_h	I.P. / E_h	χ / E_h	η / E_h
0.0000	$-\frac{1}{2}$	-460.178605	0	-460.315301	-1	-459.700969	0.136696	0.477636	0.307166	0.170470
0.0001	$-\frac{1}{2}$	-460.178705	0	-460.315301	-1	-459.701119	0.136596	0.477586	0.307091	0.170495
0.0010	$-\frac{1}{2}$	-460.179603	0	-460.315298	-1	-459.702468	0.135695	0.477136	0.306415	0.170720
0.0100	$-\frac{1}{2}$	-460.188382	0	-460.314969	-1	-459.715793	0.126588	0.472589	0.299588	0.173001
0.0200	$-\frac{1}{2}$	-460.197711	0	-460.313975	-1	-459.730264	0.116264	0.467447	0.291855	0.175592
0.0400	$-\frac{1}{2}$	-460.215030	0	-460.310012	-1	-459.758149	0.094982	0.456881	0.275932	0.180949
0.0600	$-\frac{1}{2}$	-460.230568	0	-460.303458	-1	-459.784627	0.072890	0.445942	0.259416	0.186526
0.0800	$-\frac{1}{2}$	-460.244336	0	-460.294369	-1	-459.809701	0.050033	0.434635	0.242334	0.192301
0.1000	$-\frac{1}{2}$	-460.256347	0	-460.282809	-1	-459.833379	0.026462	0.422968	0.224715	0.198253
0.2000	$-\frac{1}{2}$	-460.290698	-1	-460.311507	-1	-459.931103	0.020809	0.359595	0.190202	0.169393
0.3000	$-\frac{3}{2}$	-460.356434	-2	-460.373369	-1	-459.995459	0.016935	0.360975	0.188955	0.172020
0.4000	$-\frac{3}{2}$	-460.470968	-2	-460.507268	-2	-460.084651	0.036300	0.386317	0.211309	0.175009
0.5000	$-\frac{5}{2}$	-460.613306	-3	-460.648530	-2	-460.221887	0.035224	0.391419	0.213321	0.178098
0.6000	$-\frac{5}{2}$	-460.761533	-3	-460.811814	-2	-460.331888	0.050282	0.429645	0.239963	0.189682
0.7000	$-\frac{7}{2}$	-460.907048	-4	-460.948609	-3	-460.539395	0.041561	0.367653	0.204607	0.163046
0.8000	$-\frac{7}{2}$	-461.149040	-4	-461.177314	-3	-460.743960	0.028275	0.405080	0.216677	0.188403
0.9000	$-\frac{7}{2}$	-461.369449	-4	-461.404475	-3	-460.954316	0.035026	0.415133	0.225080	0.190054
1.0000	$-\frac{7}{2}$	-461.600106	-4	-461.649095	-3	-461.233205	0.048989	0.366901	0.207945	0.158956

TABLE XVII. Chlorine Data

**B** / B_0	M_s	E_0 / E_h	M_s^-	E_0^- / E_h	M_s^+	E_0^+ / E_h	E.A. / E_h	I.P. / E_h	χ / E_h	η / E_h
0.0000	0	-527.568715	$-\frac{1}{2}$	-527.546953	$-\frac{1}{2}$	-526.986937	-0.021762	0.581777	0.280008	0.301769
0.0001	0	-527.568715	$-\frac{1}{2}$	-527.547003	$-\frac{1}{2}$	-526.987037	-0.021712	0.581677	0.279983	0.301694
0.0010	0	-527.568712	$-\frac{1}{2}$	-527.547446	$-\frac{1}{2}$	-526.987936	-0.021267	0.580777	0.279755	0.301022
0.0100	0	-527.568497	$-\frac{1}{2}$	-527.551216	$-\frac{1}{2}$	-526.996775	-0.017281	0.571723	0.277221	0.294502
0.0200	0	-527.567845	$-\frac{1}{2}$	-527.552016	$-\frac{1}{2}$	-527.006286	-0.015829	0.561559	0.272865	0.288694
0.0400	0	-527.565238	$-\frac{1}{2}$	-527.563322	$-\frac{1}{2}$	-527.024331	-0.001917	0.540907	0.269495	0.271412
0.0600	0	-527.560898	$-\frac{1}{2}$	-527.558750	$-\frac{1}{2}$	-527.041075	-0.002148	0.519823	0.258838	0.260986
0.0800	0	-527.554832	$-\frac{1}{2}$	-527.547940	$-\frac{1}{2}$	-527.056520	-0.006893	0.498312	0.245710	0.252602
0.1000	0	-527.547050	$-\frac{1}{2}$	-527.535264	$-\frac{1}{2}$	-527.070670	-0.011785	0.476379	0.232297	0.244082
0.2000	0	-527.482865	$-\frac{1}{2}$	-527.493195	$-\frac{1}{2}$	-527.122195	0.010330	0.360669	0.185500	0.175169
0.3000	-1	-527.511555	$-\frac{3}{2}$	-527.506828	$-\frac{1}{2}$	-527.142405	-0.004727	0.369151	0.182212	0.186939
0.4000	-1	-527.588998	$-\frac{3}{2}$	-527.599206	$-\frac{3}{2}$	-527.219049	0.010209	0.369949	0.190079	0.179870
0.5000	-2	-527.751639	$-\frac{5}{2}$	-527.774061	$-\frac{3}{2}$	-527.352914	0.022421	0.398726	0.210574	0.188152
0.6000	-2	-527.897420	$-\frac{5}{2}$	-527.927654	$-\frac{5}{2}$	-527.480745	0.030235	0.416674	0.223454	0.193220
0.7000	-2	-528.013780	$-\frac{3}{2}$	-528.057010	$-\frac{5}{2}$	-527.660966	0.043231	0.352814	0.198022	0.154792
0.8000	-3	-528.201258	$-\frac{7}{2}$	-528.227406	$-\frac{5}{2}$	-527.816790	0.026148	0.384468	0.205308	0.179160
0.9000	-3	-528.369282	$-\frac{7}{2}$	-528.392876	$-\frac{5}{2}$	-527.949792	0.023595	0.419490	0.221542	0.197948
1.0000	-3	-528.516172	$-\frac{7}{2}$	-528.542749	$-\frac{7}{2}$	-528.200649	0.026577	0.315523	0.171050	0.144473

TABLE XVIII. Argon Data

**B** / B_0	M_s	E_0 / E_h	M_s^-	E_0^- / E_h	M_s^+	E_0^+ / E_h	E.A. / E_h	I.P. / E_h	χ / E_h	η / E_h
0.0000	$-\frac{1}{2}$	-1924.843620	-1	-1924.865315	0	-1924.626308	0.021695	0.217312	0.119504	0.097809
0.0001	$-\frac{1}{2}$	-1924.843719	-1	-1924.865414	0	-1924.626308	0.021695	0.217411	0.119553	0.097858
0.0010	$-\frac{1}{2}$	-1924.844616	-1	-1924.866296	0	-1924.626306	0.021680	0.218310	0.119995	0.098315
0.0100	$-\frac{1}{2}$	-1924.853265	-1	-1924.876403	0	-1924.626102	0.023138	0.227163	0.125151	0.102013
0.0200	$-\frac{1}{2}$	-1924.862204	-1	-1924.888287	0	-1924.625484	0.026083	0.236720	0.131402	0.105319
0.0400	$-\frac{1}{2}$	-1924.877982	-1	-1924.910089	0	-1924.623011	0.032107	0.254971	0.143539	0.111432
0.0600	$-\frac{1}{2}$	-1924.891013	-1	-1924.928535	0	-1924.618896	0.037522	0.272117	0.154820	0.117298
0.0800	$-\frac{1}{2}$	-1924.901376	-1	-1924.943606	0	-1924.613146	0.042230	0.288230	0.165230	0.123000
0.1000	$-\frac{1}{2}$	-1924.909159	-1	-1924.955488	0	-1924.605770	0.046329	0.303389	0.174859	0.128530
0.2000	$-\frac{3}{2}$	-1924.952137	-2	-1924.992409	-1	-1924.619611	0.040272	0.332526	0.186399	0.146127
0.3000	$-\frac{3}{2}$	-1925.009156	-2	-1925.077415	-1	-1924.656708	0.068259	0.352448	0.210354	0.142095
0.4000	$-\frac{3}{2}$	-1925.021380	-2	-1925.110601	-1	-1924.655068	0.089221	0.366312	0.227767	0.138546
0.5000	$-\frac{5}{2}$	-1925.215151	-3	-1925.312982	-2	-1924.796694	0.097831	0.418457	0.258144	0.160313
0.6000	$-\frac{5}{2}$	-1925.379507	-3	-1925.502328	-2	-1924.922373	0.122821	0.457134	0.289978	0.167157
0.7000	$-\frac{5}{2}$	-1925.506975	-3	-1925.654249	-3	-1925.035646	0.147274	0.471329	0.309302	0.162028
0.8000	$-\frac{7}{2}$	-1925.742174	-4	-1925.840112	-3	-1925.249919	0.097938	0.492255	0.295097	0.197159
0.9000	$-\frac{7}{2}$	-1925.998900	-4	-1926.092443	-3	-1925.461570	0.093543	0.537330	0.315437	0.221894
1.0000	$-\frac{7}{2}$	-1926.226069	-4	-1926.327433	-3	-1925.647781	0.101364	0.578288	0.339826	0.238462

TABLE XIX. Galium Data

**B** / B_0	M_s	E_0 / E_h	M_s^-	E_0^- / E_h	M_s^+	E_0^+ / E_h	E.A. / E_h	I.P. / E_h	χ / E_h	η / E_h
0.0000	-1	-2076.937012	$-\frac{3}{2}$	-2076.990754	$-\frac{1}{2}$	-2076.644343	0.053742	0.292669	0.173206	0.119464
0.0001	-1	-2076.937162	$-\frac{3}{2}$	-2076.990904	$-\frac{1}{2}$	-2076.644443	0.053742	0.292719	0.173231	0.119489
0.0010	-1	-2076.938509	$-\frac{3}{2}$	-2076.992248	$-\frac{1}{2}$	-2076.645341	0.053739	0.293168	0.173454	0.119715
0.0100	-1	-2076.951687	$-\frac{3}{2}$	-2077.005154	$-\frac{1}{2}$	-2076.654095	0.053467	0.297592	0.175530	0.122063
0.0200	-1	-2076.965712	$-\frac{3}{2}$	-2077.018390	$-\frac{1}{2}$	-2076.663350	0.052678	0.302362	0.177520	0.124842
0.0400	-1	-2076.991821	$-\frac{3}{2}$	-2077.041646	$-\frac{1}{2}$	-2076.680373	0.049825	0.311448	0.180637	0.130812
0.0600	-1	-2077.015366	$-\frac{3}{2}$	-2077.060902	$-\frac{1}{2}$	-2076.695420	0.045536	0.319946	0.182741	0.137205
0.0800	-1	-2077.036388	$-\frac{3}{2}$	-2077.076407	$-\frac{1}{2}$	-2076.708504	0.040019	0.327884	0.183952	0.143933
0.1000	-1	-2077.054935	$-\frac{3}{2}$	-2077.088382	$-\frac{1}{2}$	-2076.719644	0.033447	0.335291	0.184369	0.150922
0.2000	-1	-2077.112504	$-\frac{3}{2}$	-2077.148785	$-\frac{1}{2}$	-2076.746998	0.036281	0.365506	0.200894	0.164613
0.3000	-2	-2077.131924	$-\frac{5}{2}$	-2077.185044	$-\frac{3}{2}$	-2076.814509	0.053120	0.317415	0.185268	0.132148
0.4000	-2	-2077.231287	$-\frac{5}{2}$	-2077.294315	$-\frac{3}{2}$	-2076.863398	0.063028	0.367889	0.215459	0.152431
0.5000	-2	-2077.287078	$-\frac{5}{2}$	-2077.375338	$-\frac{3}{2}$	-2076.876005	0.088260	0.411073	0.249667	0.161407
0.6000	-2	-2077.304281	$-\frac{5}{2}$	-2077.419168	$-\frac{3}{2}$	-2076.855067	0.114887	0.449214	0.282051	0.167164
0.7000	-3	-2077.467197	$-\frac{7}{2}$	-2077.564946	$-\frac{5}{2}$	-2077.025065	0.097749	0.442132	0.269941	0.172192
0.8000	-3	-2077.660377	$-\frac{7}{2}$	-2077.756052	$-\frac{5}{2}$	-2077.174690	0.095675	0.485687	0.290681	0.195006
0.9000	-3	-2077.822262	$-\frac{7}{2}$	-2077.913971	$-\frac{5}{2}$	-2077.293538	0.091709	0.528724	0.310217	0.218508
1.0000	-3	-2077.955251	$-\frac{7}{2}$	-2078.045479	$-\frac{5}{2}$	-2077.383706	0.090228	0.571545	0.330887	0.240659

TABLE XX. Germanium Data

$\mathbf{B}$ / B_0	M_s	E_0 / E_h	M_s^-	E_0^- / E_h	M_s^+	E_0^+ / E_h	E.A. / E_h	I.P. / E_h	χ / E_h	η / E_h
0.0000	$-\frac{3}{2}$	-2235.814643	-1	-2235.842629	-1	-2235.447290	0.027986	0.367353	0.197670	0.169684
0.0001	$-\frac{3}{2}$	-2235.814793	-1	-2235.842779	-1	-2235.447440	0.027986	0.367353	0.197670	0.169684
0.0010	$-\frac{3}{2}$	-2235.816140	-1	-2235.844122	-1	-2235.448788	0.027982	0.367352	0.197667	0.169685
0.0100	$-\frac{3}{2}$	-2235.829304	-1	-2235.856974	-1	-2235.462049	0.027670	0.367255	0.197463	0.169793
0.0200	$-\frac{3}{2}$	-2235.843285	-1	-2235.870093	-1	-2235.476326	0.026808	0.366959	0.196884	0.170076
0.0400	$-\frac{3}{2}$	-2235.869219	-1	-2235.893163	-1	-2235.503436	0.023944	0.365783	0.194864	0.170920
0.0600	$-\frac{3}{2}$	-2235.892463	-1	-2235.912313	-1	-2235.528625	0.019850	0.363838	0.191844	0.171994
0.0800	$-\frac{3}{2}$	-2235.913047	-1	-2235.927652	-1	-2235.551902	0.014605	0.361145	0.187875	0.173270
0.1000	$-\frac{3}{2}$	-2235.931009	-1	-2235.939344	-1	-2235.573280	0.008335	0.357729	0.183032	0.174697
0.2000	$-\frac{3}{2}$	-2235.983126	-2	-2236.020033	-1	-2235.652264	0.036907	0.330862	0.183885	0.146978
0.3000	$-\frac{3}{2}$	-2236.010740	-2	-2236.057145	-1	-2235.686711	0.046405	0.324029	0.185217	0.138812
0.4000	$-\frac{3}{2}$	-2236.059877	-2	-2236.116574	-2	-2235.690994	0.056697	0.368883	0.212790	0.156093
0.5000	$-\frac{5}{2}$	-2236.140925	-3	-2236.237678	-2	-2235.799481	0.096753	0.341444	0.219099	0.122346
0.6000	$-\frac{5}{2}$	-2236.258613	-3	-2236.357511	-2	-2235.870184	0.098898	0.388429	0.243664	0.144766
0.7000	$-\frac{5}{2}$	-2236.339574	-3	-2236.439119	-2	-2235.906092	0.099545	0.433482	0.266514	0.166969
0.8000	$-\frac{5}{2}$	-2236.385695	-3	-2236.484106	-2	-2235.909491	0.098411	0.476204	0.287308	0.188897
0.9000	$-\frac{7}{2}$	-2236.441752	-4	-2236.527513	-3	-2235.975978	0.085761	0.465774	0.275768	0.190007
1.0000	$-\frac{7}{2}$	-2236.646849	-4	-2236.745755	-3	-2236.173705	0.098906	0.473144	0.286025	0.187119

TABLE XXI. Arsenic Data

$\mathbf{B}$ / B_0	M_s	E_0 / E_h	M_s^-	E_0^- / E_h	M_s^+	E_0^+ / E_h	E.A. / E_h	I.P. / E_h	χ / E_h	η / E_h
0.0000	-1	-2401.470644	$-\frac{1}{2}$	-2401.548838	$-\frac{3}{2}$	-2401.118833	0.078194	0.351811	0.215003	0.136809
0.0001	-1	-2401.470794	$-\frac{1}{2}$	-2401.548938	$-\frac{3}{2}$	-2401.118983	0.078144	0.351811	0.214978	0.136834
0.0010	-1	-2401.472140	$-\frac{1}{2}$	-2401.549833	$-\frac{3}{2}$	-2401.120331	0.077693	0.351809	0.214751	0.137058
0.0100	-1	-2401.485293	$-\frac{1}{2}$	-2401.556426	$-\frac{3}{2}$	-2401.133576	0.071133	0.351717	0.211425	0.140292
0.0200	-1	-2401.499239	$-\frac{1}{2}$	-2401.566929	$-\frac{3}{2}$	-2401.147802	0.067690	0.351437	0.209564	0.141874
0.0400	-1	-2401.525029	$-\frac{1}{2}$	-2401.581254	$-\frac{3}{2}$	-2401.174710	0.056225	0.350319	0.203272	0.147047
0.0600	-1	-2401.548032	$-\frac{1}{2}$	-2401.591921	$-\frac{3}{2}$	-2401.199562	0.043889	0.348470	0.196180	0.152291
0.0800	-1	-2401.568272	$-\frac{1}{2}$	-2401.599051	$-\frac{3}{2}$	-2401.222365	0.030779	0.345907	0.188343	0.157564
0.1000	-1	-2401.585782	$-\frac{1}{2}$	-2401.602761	$-\frac{3}{2}$	-2401.243131	0.016979	0.342651	0.179815	0.162836
0.2000	-1	-2401.633856	$-\frac{3}{2}$	-2401.663944	$-\frac{3}{2}$	-2401.316929	0.030088	0.316927	0.173508	0.143420
0.3000	-2	-2401.682321	$-\frac{5}{2}$	-2401.711466	$-\frac{3}{2}$	-2401.342514	0.029145	0.339807	0.184476	0.155331
0.4000	-2	-2401.725877	$-\frac{5}{2}$	-2401.767229	$-\frac{3}{2}$	-2401.349953	0.041352	0.375924	0.208638	0.167286
0.5000	-2	-2401.740770	$-\frac{5}{2}$	-2401.841223	$-\frac{3}{2}$	-2401.405593	0.100453	0.335177	0.217815	0.117362
0.6000	-2	-2401.802495	$-\frac{5}{2}$	-2401.908328	$-\frac{5}{2}$	-2401.447355	0.105833	0.355140	0.230487	0.124654
0.7000	-3	-2402.027515	$-\frac{7}{2}$	-2402.099121	$-\frac{5}{2}$	-2401.580436	0.071606	0.447079	0.259343	0.187737
0.8000	-3	-2402.152208	$-\frac{7}{2}$	-2402.232590	$-\frac{5}{2}$	-2401.691831	0.080382	0.460377	0.270380	0.189998
0.9000	-3	-2402.242309	$-\frac{7}{2}$	-2402.329807	$-\frac{5}{2}$	-2401.771260	0.087498	0.471049	0.279274	0.191776
1.0000	-3	-2402.300400	$-\frac{7}{2}$	-2402.397379	$-\frac{5}{2}$	-2401.821035	0.096979	0.479365	0.288172	0.191193

TABLE XXII. Selenium Data

$\mathbf{B}$ / B_0	M_s	E_0 / E_h	M_s^-	E_0^- / E_h	M_s^+	E_0^+ / E_h	E.A. / E_h	I.P. / E_h	χ / E_h	η / E_h
0.0000	$-\frac{1}{2}$	-2574.075904	0	-2574.203548	-1	-2573.636570	0.127644	0.439334	0.283489	0.155845
0.0001	$-\frac{1}{2}$	-2574.075954	0	-2574.203547	-1	-2573.636720	0.127593	0.439234	0.283414	0.155821
0.0010	$-\frac{1}{2}$	-2574.076401	0	-2574.203543	-1	-2573.638067	0.127142	0.438334	0.282738	0.155596
0.0100	$-\frac{1}{2}$	-2574.080552	0	-2574.203093	-1	-2573.651299	0.122541	0.429253	0.275897	0.153356
0.0200	$-\frac{1}{2}$	-2574.091582	0	-2574.201730	-1	-2573.665488	0.110148	0.426094	0.268121	0.157973
0.0400	$-\frac{1}{2}$	-2574.107650	0	-2574.196300	-1	-2573.692243	0.088650	0.415407	0.252029	0.163379
0.0600	$-\frac{1}{2}$	-2574.121107	0	-2574.187314	-1	-2573.716840	0.066207	0.404267	0.235237	0.169030
0.0800	$-\frac{1}{2}$	-2574.131970	0	-2574.174846	-1	-2573.739285	0.042876	0.392685	0.217781	0.174905
0.1000	$-\frac{1}{2}$	-2574.140260	0	-2574.158981	-1	-2573.759589	0.018721	0.380671	0.199696	0.180975
0.2000	$-\frac{1}{2}$	-2574.144114	-1	-2574.174883	-1	-2573.829474	0.030769	0.314640	0.172705	0.141936
0.3000	$-\frac{3}{2}$	-2574.188087	-2	-2574.217053	-1	-2573.848352	0.028966	0.339735	0.184351	0.155385
0.4000	$-\frac{3}{2}$	-2574.225447	-2	-2574.269923	-2	-2573.883567	0.044476	0.341880	0.193178	0.148702
0.5000	$-\frac{5}{2}$	-2574.287473	-3	-2574.373022	-2	-2573.931177	0.085549	0.356296	0.220923	0.135374
0.6000	$-\frac{5}{2}$	-2574.326931	-3	-2574.432545	-2	-2573.935797	0.105614	0.391134	0.248374	0.142760
0.7000	$-\frac{7}{2}$	-2574.442164	-4	-2574.495490	-3	-2573.996559	0.053326	0.445605	0.249466	0.196140
0.8000	$-\frac{7}{2}$	-2574.561448	-4	-2574.629575	-3	-2574.191977	0.068127	0.369471	0.218799	0.150672
0.9000	$-\frac{7}{2}$	-2574.733988	-4	-2574.820838	-3	-2574.353210	0.086850	0.380778	0.233814	0.146964
1.0000	$-\frac{7}{2}$	-2574.887101	-4	-2574.983951	-3	-2574.482260	0.096850	0.404841	0.250846	0.153996

TABLE XXIII. Bromine Data

$\mathbf{B}$ / B_0	M_s	E_0 / E_h	M_s^-	E_0^- / E_h	M_s^+	E_0^+ / E_h	E.A. / E_h	I.P. / E_h	χ / E_h	η / E_h
0.0000	0	-2753.710339	$-\frac{1}{2}$	-2753.692673	$-\frac{1}{2}$	-2753.192917	-0.017666	0.517422	0.249878	0.267544
0.0001	0	-2753.710339	$-\frac{1}{2}$	-2753.692723	$-\frac{1}{2}$	-2753.192967	-0.017616	0.517372	0.249878	0.267494
0.0010	0	-2753.710336	$-\frac{1}{2}$	-2753.693163	$-\frac{1}{2}$	-2753.193415	-0.017173	0.516921	0.249874	0.267047
0.0100	0	-2753.710010	$-\frac{1}{2}$	-2753.696731	$-\frac{1}{2}$	-2753.197641	-0.013279	0.512369	0.249545	0.262824
0.0200	0	-2753.709025	$-\frac{1}{2}$	-2753.702775	$-\frac{1}{2}$	-2753.208891	-0.006250	0.500134	0.246942	0.253192
0.0400	0	-2753.705084	$-\frac{1}{2}$	-2753.704601	$-\frac{1}{2}$	-2753.225782	-0.000483	0.479302	0.239410	0.239893
0.0600	0	-2753.698524	$-\frac{1}{2}$	-2753.695467	$-\frac{1}{2}$	-2753.240603	-0.003057	0.457921	0.227432	0.230489
0.0800	0	-2753.689357	$-\frac{1}{2}$	-2753.681476	$-\frac{1}{2}$	-2753.253359	-0.007881	0.435998	0.214059	0.221940
0.1000	0	-2753.677600	$-\frac{1}{2}$	-2753.665550	$-\frac{1}{2}$	-2753.264059	-0.012050	0.413541	0.200746	0.212796
0.2000	0	-2753.580765	$-\frac{1}{2}$	-2753.601550	$-\frac{1}{2}$	-2753.287054	0.020785	0.293711	0.157248	0.136463
0.3000	-1	-2753.616726	$-\frac{3}{2}$	-2753.631321	$-\frac{1}{2}$	-2753.260522	0.014595	0.356204	0.185400	0.170805
0.4000	-2	-2753.653267	$-\frac{5}{2}$	-2753.701419	$-\frac{3}{2}$	-2753.315925	0.048152	0.337342	0.192747	0.144595
0.5000	-2	-2753.727809	$-\frac{5}{2}$	-2753.796651	$-\frac{3}{2}$	-2753.356142	0.068842	0.371667	0.220255	0.151413
0.6000	-2	-2753.752237	$-\frac{5}{2}$	-2753.846770	$-\frac{5}{2}$	-2753.400408	0.094533	0.351829	0.223181	0.128648
0.7000	-3	-2753.892323	$-\frac{7}{2}$	-2753.936320	$-\frac{5}{2}$	-2753.450042	0.043997	0.442281	0.243139	0.199142
0.8000	-3	-2753.953774	$-\frac{7}{2}$	-2754.007754	$-\frac{7}{2}$	-2753.527515	0.053980	0.426259	0.240120	0.186140
0.9000	-3	-2753.994867	$-\frac{7}{2}$	-2754.058453	$-\frac{7}{2}$	-2753.675887	0.063586	0.318980	0.191283	0.127697
1.0000	-3	-2754.088605	$-\frac{7}{2}$	-2754.165534	$-\frac{7}{2}$	-2753.789943	0.076929	0.298662	0.187796	0.110867

TABLE XXIV. Krypton Data
